# Two-Dimensional Materials for Highly Efficient and Stable Perovskite Solar Cells

**DOI:** 10.1007/s40820-024-01417-1

**Published:** 2024-05-23

**Authors:** Xiangqian Shen, Xuesong Lin, Yong Peng, Yiqiang Zhang, Fei Long, Qifeng Han, Yanbo Wang, Liyuan Han

**Affiliations:** 1 State Key Laboratory of Metal Matrix Composites, Shanghai Jiao Tong University, Shanghai, 200240 People’s Republic of China; 2 Xinjiang Key Laboratory of Solid State Physics and Devices, School of Physical Science and Technology, Xinjiang University, Urumqi, 830046 People’s Republic of China; 3 Special Division of Environmental and Energy Science, College of Arts and Sciences, Komaba Organization for Educational Excellence, University of Tokyo, Tokyo, 153-8902 Japan; 4 State Key Laboratory of Advanced Technology for Materials Synthesis and Processing, Wuhan University of Technology, Wuhan, 430070 People’s Republic of China; 5 College of Chemistry, Henan Institute of Advanced Technology, Zhengzhou University, Zhengzhou, 450001 People’s Republic of China; 6 Guangxi Key Laboratory of Optical and Electronic Materials and Devices, Collaborative Innovation Center for Exploration of Nonferrous Metal Deposits and Efficient Utilization of Resources, School of Materials Science and Engineering, Guilin University of Technology, Guilin, 541004 People’s Republic of China

**Keywords:** Perovskite solar cells, Two-dimensional materials, Interface engineering, Van der Waals heterojunction, Electrodes

## Abstract

Perovskite solar cells (PSCs) offer low costs and high power conversion efficiency. However, the lack of long-term stability, primarily stemming from the interfacial defects and the susceptible metal electrodes, hinders their practical application. In the past few years, two-dimensional (2D) materials (e.g., graphene and its derivatives, transitional metal dichalcogenides, MXenes, and black phosphorus) have been identified as a promising solution to solving these problems because of their dangling bond-free surfaces, layer-dependent electronic band structures, tunable functional groups, and inherent compactness. Here, recent progress of 2D material toward efficient and stable PSCs is summarized, including its role as both interface materials and electrodes. We discuss their beneficial effects on perovskite growth, energy level alignment, defect passivation, as well as blocking external stimulus. In particular, the unique properties of 2D materials to form van der Waals heterojunction at the bottom interface are emphasized. Finally, perspectives on the further development of PSCs using 2D materials are provided, such as designing high-quality van der Waals heterojunction, enhancing the uniformity and coverage of 2D nanosheets, and developing new 2D materials-based electrodes.

## Highlights

- Recent progress on the applications of 2D materials in perovskite solar cells is discussed from the views of bottom interfaces, top interfaces, and electrodes.
- The roles of van der Waals heterojunction in enhancing the performance of perovskite solar cells are highlighted.
- The future directions and challenges in development of 2D materials-based perovskite solar cells are provided.

## Graphical abstract

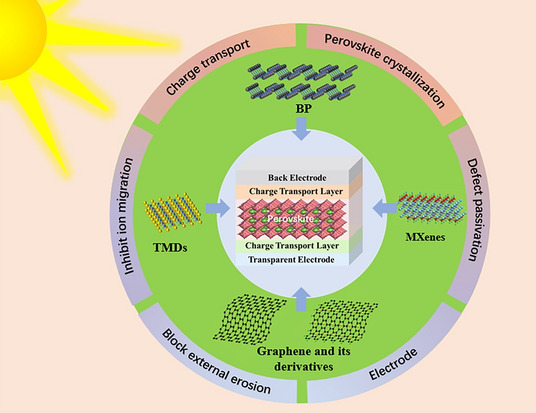

## Introduction

In the past decade, perovskite solar cells (PSCs) have attracted a lot of attention due to their excellent optoelectronic properties, low cost, and facile manufacturing process [1–7]. Researchers have dedicated efforts to enhancing the power conversion efficiency (PCE) and stability of PSCs through various strategies such as architecture design [8, 9], composition engineering [5, 10], charge transport layer (CTL) optimization [11, 12], defect passivation [6, 13], and interface modification [14, 15]. Notably, the state-of-the-art PSCs achieved a certified PCE exceeding 26%, which is comparable to that of monocrystalline silicon solar cells [16–19]. However, there are still challenges that need to be overcome for commercialization, including the notorious interfaces and unsatisfactory electrodes. These issues hinder the progress of PCE in reaching the Shockley–Queisser limit and maintaining long-term stability under external environmental conditions [20–23].

In the case of a typical PSC, the interfaces are composed of electrodes, CTLs, and perovskite [24, 25]. Due to the incompatibility between materials in terms of crystal lattice, thermal expansion coefficient, energy level, and carrier mobility, these interfaces are characterized by a high defect state density and low ion migration barriers [21, 26, 27]. Furthermore, the solution-processed polycrystalline perovskite, along with its ionic nature, makes these issues even more severe [28–31]. Therefore, the carrier accumulation, non-radiative recombination, and degradation reactions usually occur at the interfaces of PSCs, which leads to a decrease in both device efficiency and stability [32–35]. In addition, the durability of PSCs is constrained by the traditional metal electrodes that are susceptible to the migrated ions from the perovskite [22, 36]. As for the flexible PSCs, the poor mechanical stability of the transparent conductive oxides is also a weakness [37, 38]. Therefore, there is an urgent need to improve the interface quality and develop new electrode materials to realize efficient and stable PSCs.

Two-dimensional (2D) materials are ultrathin materials that can be only a few atoms thick, providing unique properties that are different from their bulk counterparts [39, 40]. In the past few years, various 2D materials, including graphene and its derivatives, transitional metal dichalcogenides (TMDs), transitional metal carbides and/or nitrides (MXenes), and black phosphorus (BP) have been developed and demonstrated exceptional performance in fields such as transistors, sensors, biomedicine, catalysts, and photovoltaic cells [41–45]. The first application of 2D materials in PSCs can be traced back to 2013 when H.J. Snaith and colleagues enhanced the PCE of mesoporous PSCs from 10.0% to 15.6% by modifying the TiO_2_ electron transport layer (ETL) using graphene nanoflakes [46]. This enhancement is attributed to the exceptional conductivity of graphene and its optimal work function (WF) situated between the conduction bands of FTO and TiO_2_, which provide a highway for electron transportation and collection.

**Fig. 1:**
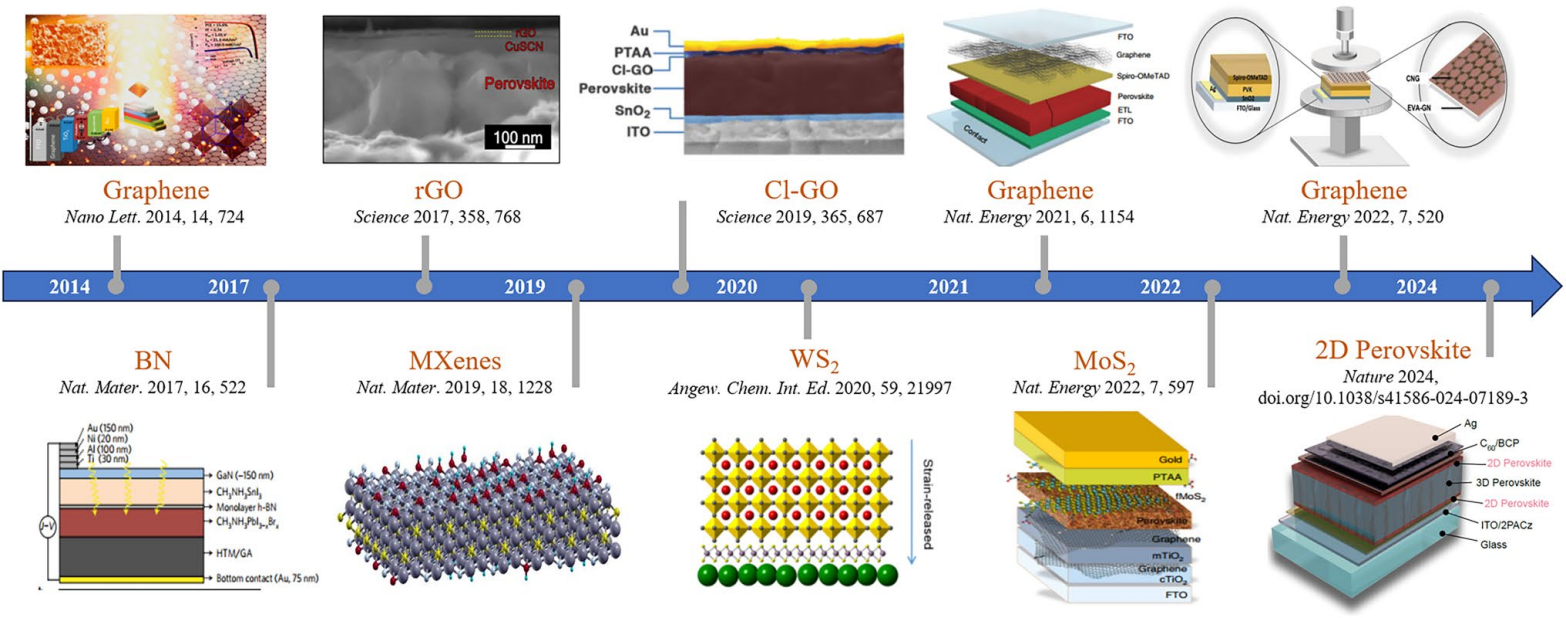
The key milestones of 2D materials-incorporated PSCs over the past decade. Reproduced with permission from Ref. [46]. Copyright 2013, American Chemical Society. Reproduced with permission from Ref. [47]. Copyright 2016, Springer Nature. Reproduced with permission from Ref. [48]. Copyright 2017, AAAS. Reproduced with permission from Ref. [49]. Copyright 2019, Springer Nature. Reproduced with permission from Ref. [50]. Copyright 2019, AAAS. Reproduced with permission from Ref. [51]. Copyright 2020, Wiley. Reproduced with permission from Ref. [52]. Copyright 2021, Springer Nature. Reproduced with permission from Ref. [53]. Copyright 2022, Springer Nature. Reproduced with permission from Ref. [36]. Copyright 2022, Springer Nature. Reproduced with permission from Ref. [8]. Copyright 2024, Springer Nature

Soon thereafter, researchers recognized that 2D materials are desirable for use as the interface and electrode materials in PSCs, mainly due to the following advantages: firstly, 2D materials are notable for their atomic-level thickness, smooth surfaces, and lack of dangling bonds [54]. Unlike traditional interface contacts, 2D materials form van der Waals (vdW) heterostructures through physical adsorption with other materials, thereby minimizing the formation of defects at the interface [55]. This unique interaction has been shown to facilitate the epitaxial growth of perovskite thin films, optimizing their crystallization and orientation [51, 56]. Secondly, the electronic structure of 2D materials, including their band gap and WF, is significantly influenced by their atomic layers and surface functional groups. This offers the opportunity for precise manipulation of interface barriers, minimizing the energy loss during carrier extraction and transport across the interfaces [57, 58]. Thirdly, due to the superior conductivity, some of 2D materials can function as the electrode to realize efficient charge collection [59, 60]. Moreover, 2D materials possess excellent chemical and mechanical stability with high compactness, which render them resistant to moisture, oxygen, ion migration, and metal diffusion. Based on all these benefits, 2D materials successfully find their applications in perovskite crystallization, charge transport, defect passivation, and electrode (Fig. 1) [45, 61–63].

In this review, recent advancements in the incorporation of 2D materials into PSCs are summarized. To gain a deeper understanding of 2D materials and the interfaces they form, we first present a brief introduction to the fundamental properties of 2D materials and vdW heterostructures based on them. Then, we focus on the applications of 2D materials at the bottom interfaces, top interfaces, and electrodes of PSCs, respectively. We also delve into the underlying mechanisms that contribute to improved device efficiency and stability. At the end of this review, the conclusions and possible pathways toward unlocking the full potential of 2D materials-incorporated PSCs are outlined.

## 2D Materials and Van der Waals Heterojunctions

2D materials are defined as nanoscale materials ranging from 1 to 100 nm, which derive into a wide range of electronic properties including conductors (metal and semi-metal), semiconductors (direct and indirect bandgap), and insulators [39, 56, 57]. The confinement of carrier mobility gives rise to a plethora of peculiar properties and applications, such as bandgap tunability for photoelectric devices, spin controllability for spintronics, and structure anisotropy for various polarized devices [54, 64, 65]. Furthermore, the integration of 2D materials with other functional layers is a vital approach for forming semiconductor heterojunctions or electric contacts, which can integrate diverse properties into modern optoelectronic devices. Notably, vdW heterojunctions can be physically assembled through weak vdW interactions, without limitations in regard to lattice matching and processing compatibility. This significant advantage enables the extensive applications of 2D materials on the photovoltaics interfaces [55, 66]. In Sect. 2, we summarize the category of 2D materials and analyze their basic properties, as well as discuss the vdW heterojunctions of 2D materials.

### Category and Basic Properties of 2D Materials

Currently, the four most commonly utilized 2D materials in PSCs are graphene and its derivatives, TMDs, MXenes, and BP [45, 57, 61]. Besides, other 2D materials such as antimonene, hexagonal boron nitride (h-BN), metal-free carbon nitrides (CNs), metal–organic frameworks (MOFs), and 2D perovskite materials have also been reported in PSCs [67–71]. The basic properties of typical examples are summarized in Table 1.

**Table 1:** Basic properties of representative 2D materials

2D materials	Bandgap (eV)	Band type	Carrier mobility (cm^2^ V^−1^ s^−1^)	Refs
Graphene	0	Direct	10^3^ to 2 × 10^5^	[79]
GO	2.00	Indirect	N/A	[80, 81]
rGO	0.02 to 2.00	Indirect to direct	1.2 × 10^2^	[82]
*h*-BN	5.90	Direct	1	[83]
MoS_2_	1.72	Direct	10 to 1.3 × 10^2^	[44, 84]
Ti_3_C_2_T_X_	1.05	Direct	3.4 × 10	[85]
Phosphorene	1.67	Direct	7 × 10^2^ to 2.6 × 10^4^	[86]
BP	1.60	Direct	10^4^ to 2.6 × 10^4^	[87]
Antimonene	1.75	Indirect	10^5^ to 4 × 10^5^	[88]

The graphene and its derivatives possess atomic layers arranged in a hexagonal honeycomb lattice. As the first investigated 2D material, graphene is an atomically thin *sp*^2^ carbon layer arranged in a hexagonal honeycomb lattice [40]. As reported by previous works [72, 73], the mechanical stability of the 2D materials described as cohesive energy is inversely proportional to the bond length. Among the 2D materials, high-symmetry graphene shows the shortest bond length, making it the most stable 2D material known to date. The electronic bands near the six corners of the 2D hexagonal Brillouin zone cross the Fermi level, resulting in a linear alteration of electron energy of a band within the Brillouin zone at these points. This unique feature gives rise to a zero effective mass for carriers and imparts graphene with semi-metal properties, ultimately endowing it with the highest recorded electron mobility up to 2 × 10^5^ cm^2^ V^−1^ s^−1^ at finite temperatures [39, 74]. The electronic structure engineering of graphene has rapidly progressed toward developing semiconducting modifications that exhibit an extremely low bandgap, thus paving the way for photovoltaics and electronic applications [39]. Nonetheless, these devices call for a requirement of larger bandgap semiconductors and insulators. Recently, researchers found that the properties of graphene and its derivatives, including the bandgap, conductivity, and dispersibility, can be precisely tuned through the regulation of functional groups and layer numbers [75, 76]. For example, the introduction of various groups to the graphene surface, such as epoxide, carbonyl, and hydroxyl groups, can yield GO [77]. The further chemical reduction process can transform GO into reduced GO (rGO). Unlike GO, which is an insulator, rGO displays a high conductivity while retaining the promising dispersibility of GO [78].

TMDs are a new star in the post-graphene era, which are composed of a transition metal and two chalcogens. The general stoichiometric formula of TMDs is MX_2_, where M and X denoted the transition metal (Ti, V, Ta, Mo, W, Re, etc.) and chalcogen (S, Se, Te, etc.), respectively [89]. The structure of TMDs is layered with the transition metal sandwiched between two chalcogens, which are bound together by weak vdW forces that make it easy to be exfoliate into single or few layers. TMDs have a variety of electronic band structures, covering materials such as conductors, semimetals, semiconductors, insulators, and superconductors [84]. Some of the well-known and widely studied TMDs include molybdenum disulfide (MoS_2_), tungsten disulfide (WS_2_), tungsten diselenide (WSe_2_), and molybdenum ditelluride (MoTe_2_) [39, 90]. Recently, the layer-dependent characteristics of TMDs have attracted widespread attention. For example, when reducing from bulk to monolayer, MoS_2_ undergoes a transition from an indirect bandgap to a direct bandgap, accompanied by an increase in bandgap energy from 1.2 eV to the range of 1.7–1.9 eV [44, 91]. In addition, monolayer MoS_2_ exhibits photoluminescence and valley polarization in its electronic structure, which opens up possibilities for use in next-generation optoelectronic devices such as light-emitting diodes, lasers, and detectors [92].

MXenes are an emerging family of 2D materials that have attracted significant attention in materials science and nanotechnology due to their unique physical and chemical properties [42]. The general formula for MXenes is M_n+1_X_n_T_x_ (n = 1–4), where M represents an early transition metal (Sc, Ti, Zr, Hf, V, Nb, Ta, Cr, Mo, etc.), X represents carbon and/or nitrogen, and T_x_ stands for surface terminations such as –O, –OH, and –F [93]. MXenes have a layered structure similar to that of graphene but with a larger interlayer distance due to the presence of surface terminations. The monolayer or multilayer morphology of MXenes is typically synthesized from M_n+1_AX_n_ (MAX) phase precursors by removing the monoatomic layers of the A element, which belongs to group 13 or 14 (e.g., Al, Ga, Si, or Ge) [94]. MXenes possess a range of remarkable properties including high electrical conductivity, good mechanical strength, and excellent chemical stability [95]. In addition, the electronic structure and WF of MXenes can be facile tailored by modifying their surface terminations. Typical MXenes include Ti_2_CT_x_, Ti_3_C_2_T_x_, Nb_2_CT_x_, and (Mo, V)_5_C_4_T_x_. Notably, Ti_3_C_2_T_x_ was first reported and is widely used in PSCs [96, 97].

BP is a layered material that consists of single or multiple layers of phosphorus atoms arranged in a honeycomb structure. It has a puckered structure resulting from the strong covalent bonds within each layer and weak vdW interactions between layers [98–100]. The intricate structure endows BP high in-plane anisotropy, meaning its electrical and optical properties vary depending on the orientation of the layer with respect to the crystalline axis. BP has a direct bandgap that can be adjusted by its thickness. One of the most exceptional features of BP is its high carrier mobility, which allows for fast charge transport [101]. Moreover, the carrier transport in BP is dominated by quantum tunneling effects, which can lead to ambipolar behavior and strong nonlinearity [102]. These attributes enable BP to simultaneously transport n-type and p-type carriers, opening up new opportunities for field-effect transistors, optoelectronics, and photovoltaic devices [99]. Furthermore, BP boasts excellent mechanical properties with high tensile strength and ductility, making it a promising material for flexible electronics and stretchable devices [43].

### Van der Waals Heterojunctions of 2D Materials

**Fig. 2:**
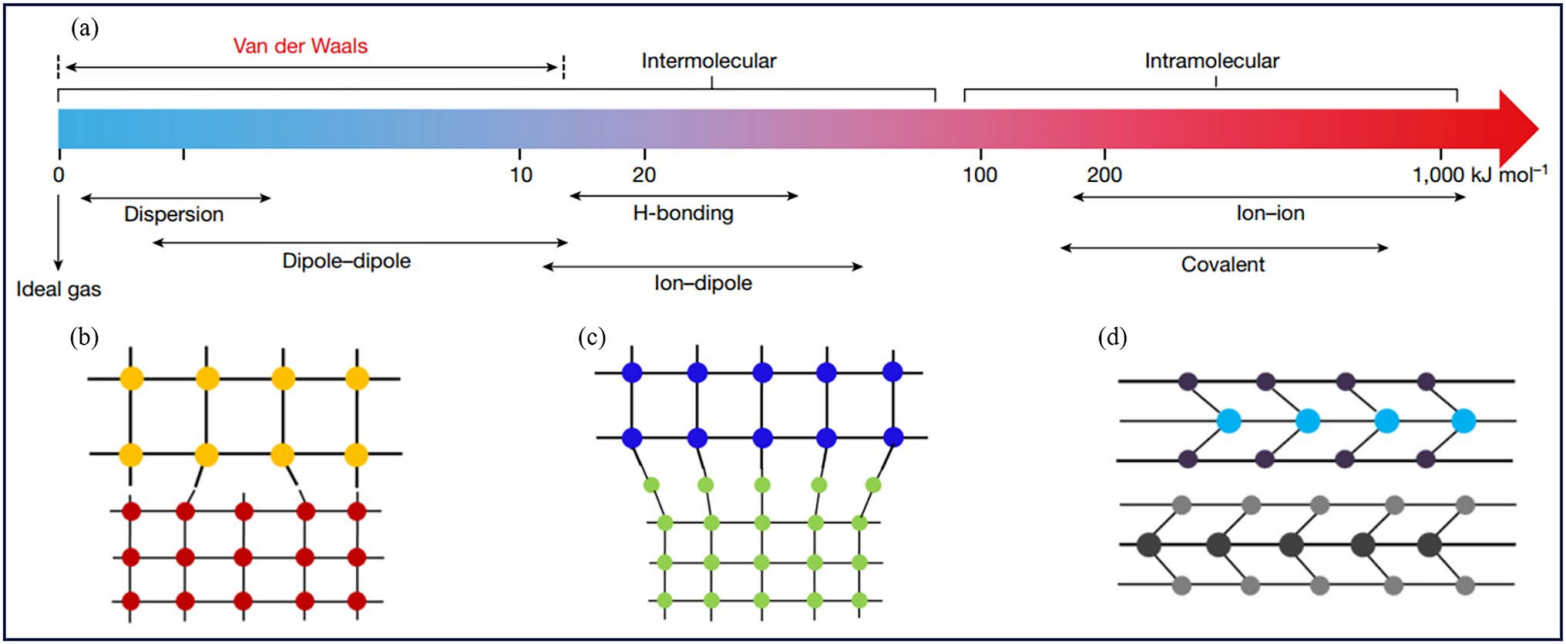
**a** Energies of various molecular interactions. The vdW interaction is the weakest intermolecular interaction (also termed physical interaction), much smaller than typical intramolecular interactions (also termed chemical interactions). Reproduced with permission from Ref. [55]. Copyright 2019, Springer Nature. **b-d** Schematic illustration of the interfaces connected by **b** covalent bonds, **c** ionic bonds, and **d** vdW interaction. Reproduced with permission from Ref. [39]. Copyright 2013, American Chemical Society

Integrating disparate materials with pristine interfaces is an indispensable step toward creating functional devices through deliberate design, which has been a longstanding objective of the material science community [54]. Currently, two dominant methods for constructing heterojunction have emerged, relying on either strong intramolecular interactions (e.g., covalent bond and ionic bond) or weak intermolecular interactions (e.g., vdW interaction) (Fig. 2a). The vdW interaction strength is typically of the order of 0.1–10 kJ mol^−1^, about 2–3 orders of magnitude smaller than that of ionic or covalent bonds (about 100–1,000 kJ mol^−1^) [55].

Although chemical bonds yield strong interfacial bonding, the success of this strategy depends on highly matched lattices and one-to-one paired bonding atoms between the surfaces of materials. Materials with lattice mismatch of greater than 5% result in a lot of dangling bonds and severe interface disorder, which could degrade intrinsic properties of the corresponding devices (Fig. 2b, c) [39, 103]. On the contrary, vdW heterojunctions are integrated via the physical assemble of pre-fabricated building blocks through weak vdW forces, thus presenting an alternative approach to low-energy material integration. This physical assembly does not rely on the direct chemical bonding and bypasses the constraints of lattice matching and compatibility of synthesis conditions (Fig. 2d) [104]. Therefore, this bond-free integration method exhibits no obvious restriction on material similarities and can be generally applied to construct feasible vdW heterojunctions between disparate materials with distinct crystal structures (e.g., crystallinity, lattice symmetry and constant), electronic properties (e.g., insulators, semiconductors, metals and superconductors), or material dimensions (e.g***.***, 0D quantum dot, 1D nanowire, 2D nanosheet, and 3D bulk phase) [105–108]. The isolation of graphene and various 2D atomic materials with surfaces free of dangling bonds has sparked significant interest in vdW integration, which enables the creation of an array of heterojunctions with atomically clean and electronically sharp interfaces. This not only provides a fertile ground for fundamental studies, but also paves the way for exciting new device concepts [109].

In PSCs, the vdW heterostructure is created by combining of 2D materials with perovskite layers, with examples including graphene [110], MoS_2_ [111], WS_2_ [51], SnS_2_ [112], WSe_2_ [58], antimonene [113], and 2D perovskite [71]. These 2D materials typically function as templates, upon which the perovskite is epitaxially grown. This approach leads to the formation of a high-quality perovskite film with the following mechanism: (i) The 2D interlayer effectively serves as a physical barrier, which screens the defects and chemical interaction between the substrate and the perovskite film. (ii) The smoother 2D materials reduce surface roughness, enabling a longer atomic diffusion length and ultimately promoting the formation of larger crystalline grains. (iii) The low interfacial energy between 2D materials and perovskites prevents perovskite deformations during its growth process, which reduces the occurrence of voids and cracks within the thin films. Additional, these heterojunctions also enable effective photogenerated carrier transport and extraction through type II or quasi-type II junction [114]. By surface functionalizing 2D materials in these heterojunctions, charged defects (e.g., under-coordinated Pb cations, free I anions, interstitials, and substitution) can be passivated at both top and bottom perovskite interfaces, consequently reducing non-radiative recombination [115, 116]. Furthermore, vdW heterojunctions provide an excellent strategy to prevent ion migration in perovskite photovoltaics; for example, a 2D/3D vdW heterojunction based on graphene possesses a lattice parameter of 0.246 nm, which is smaller than the radius of I anions (0.412 nm), and thus precludes the corrosion of functional layers induced by ion migration [117]. In conclusion, the aforementioned advantages of vdW heterojunctions hold great potential for interface modification and can enhance the efficiency and stability of ideal perovskite photovoltaics.

## 2D Materials at Bottom Interfaces

The bottom interface refers to the interface between the transparent electrode and the perovskite layer. For a regular device, the bottom interface includes the interfaces of transparent electrode/ETL and ETL/perovskite. Therefore, the main function of the bottom interface is to extract electrons from the active layer and conduct them to the transparent electrode [20]. On the other hand, the bottom interfaces of inverted device have an opposite configuration and direction of charge transport. The bottom interface, being the site for nucleation and growth of perovskite, plays a pivotal role in the formation of perovskite thin films. The crystal lattice constant, thermal expansion coefficient, and wettability of the bottom interface material directly influence the quality of the perovskite thin films, including their crystallinity, surface morphology, and optoelectronic properties [111, 118]. Moreover, since sunlight traverses the PSCs from the bottom to the top, the carrier concentration within the perovskite layer proximal to the bottom interface is higher than those present in other regions. Consequently, the bottom interface material must exhibit high carrier mobility and conductivity, alongside energy levels that are compatible with the perovskite film to prevent carrier aggregation and recombination [25, 31]. Besides, to guarantee optimal absorption and irradiation stability of the perovskite layer, it is required that the bottom interface has a high transmittance within the visible light spectrum while effectively shielding ultraviolet light [119–121]. In Sect. 3, we summarize the applications of 2D materials in enhancing the bottom interface of PSCs. The discussion focuses on their crucial role in enhancing the crystallization of perovskites, optimizing charge dynamics, and fine-tuning optical properties. Representative 2D materials at bottom interface for the enhancement of device efficiency and stability are illustrated in Table 2.

**Table 2:** Representative 2D materials at the bottom interface for improving the efficiency and stability of PSCs

2D materials	Device structure	Function	PCE (%)	Characteristic and stability	Refs
Graphene	FTO/TiO_2_/Graphene/CsPbBr_3_/Carbon	vdW epitaxial growth of CsPbBr_3_, improve the electron extraction	10.64	All-inorganic, HTL-free, 91% its initial PCE after 2,000 h in ambient air	[110]
rGO	ITO/rGO/PTAA/MAPbI_3_/PCBM/BCP/Ag	Block ultraviolet light, gradient energy level between PTAA and ITO	17.2	Active area of 1.02 cm^2^, 90% of its initial PCE after 1-sun illumination for 500 h	[78]
I-GQDs	ITO/SnO_2_/I-GQDs/FAPbI_3_/spiro-OMeTAD/Ag	Enhance the conductivity and eliminate surface defects of ETL, improve the crystallization of FAPbI_3_	22.37	Over 84% of its initial PCE after 1-sun illumination for 500 h in a nitrogen gas glove box, without encapsulation, at room temperature	[122]
I-GQDs	ITO/SnO_2_/I-GQDs/CsFAMA/spiro-OMeTAD/Ag	Passivate the defects of SnO_2_, enhance UV light utilization through down-conversion	24.11	81% of its initial PCE after continuous UV irradiation (365 nm, 20 mW cm^−2^) for 300 h	[123]
N,Cl-GQDs	ITO/PEDOT:PSS/N,Cl-GQDs /FAMA(Sn_0.6_Pb_0.4_)I_3_/PCBM/BCP/Ag	Regulate the charge distribution and passivate the defect states	21.5	90% of its initial PCE after stored in glove box for 1,000 h without encapsulation	[124]
MoS_2_	ITO/PTAA/MoS_2_/MAPbI_3_/PCBM/BCP/Ag	vdW epitaxy growth of MAPbI_3_	20.55	80% of its initial PCE after 800 h in the dark ambient with 30% RH, without encapsulation	[111]
WS_2_	FTO/SnO_2_-TiO_x_Cl_4-2x_/WS_2_/CsPbBr_3_/Carbon	WS_2_/CsPbBr_3_ vdW heterostructure, release interfacial strain	10.65	All-inorganic, HTL-free. 90% of its initial PCE after continuous illumination for 10,000 s at 100 mW cm^−2^; over 80% of its initial PCE after 120 days in ambient condition with 80% RH at 25 °C	[51]
WSe_2_	ITO/NiO_x_/WSe_2_/FASnI_3_/PCBM/BCP/Ag	vdW epitaxial growth, cascade band structure	10.47	Lead-free, 82% its initial PCE after 1,000 h in air condition with 20% RH, without encapsulation	[58]
SnS_2_	FTO/SnO_2_:SnS_2_/CsPbBr_3_/Carbon	Plays a role of “bridge” to connect incompatible interface, epitaxial growth	10.72	All-inorganic, HTL-free, 91% its initial PCE after 700 h in air condition with 80% RH and at 25 °C, without encapsulation	[112]
Ti_3_C_2_T_x_	ITO/SnO_2_-Ti_3_C_2_T_x_/CsFAMA/spiro-OMeTAD/Au	Improve the dispersion and electronic property of the SnO_2_ nanoparticles, induces a vertical growth of perovskite	23.07	90% its initial PCE after 500 h in ambient air with 30%–40% RH	[125]
Ti_3_C_2_T_x_	PEN/ITO/Ti_3_C_2_T_x_-PEDOT:PSS/CsFAMA/PCBM/BCP/Ag	Energy level alignment, facilitate charge extraction, improve the quality of perovskite film	17.06	Flexible, minimodule (15 cm^2^), 90% its initial PCE after 1,200 h in ambient air at 85 °C	[126]
Ti_3_C_2_Cl_x_	ITO/SnO_2_-Ti_3_C_2_Cl_x_/FAMAPb(I_0.95_Br_0.05_)_3_/o-TB-GDY/spiro-OMeTAD/Au	Improve the perovskite crystallization, suppress non-radiative recombination by forming the Pb-Cl bond	24.86	92% its initial PCE after 1,464 h in ambient air; 80% its initial PCE after 1,002 h of thermal stability test at 85 °C	[127]
Nb_2_CT_x_	ITO/Nb_2_CT_x_/FA_0.85_Cs_0.15_PbI_3_/spiro-OMeTAD/Ag	High conductivity, retards the crystallization process of perovskite	21.79	93% its initial PCE after 1,500 h in glovebox	[128]
BP	FTO/c-TiO_2_/mp-TiO_2_/BP-3/CsMAFAPb(I_0.83_Br_0.17_)_3_/BP-1/spiro-OMeTAD/Au	Enhance charge extraction and light absorption, suppress carrier recombination	19.83	95% its initial PCE after 180 days in air, encapsulated with a parylene film	[129]
PNRs	ITO/PTAA:PNRs/MAPbI_3_/PCBM/BCP/Cu	Enhance hole extraction, improve both the mobility and conductivity of the PTAA	21.14	N/A	[130]
Antimonene	ITO/PTAA/Antimonene/MAPbI_3_/PCBM/Bphen/Al	Enhance hole extraction and transport	20.11	N/A	[67]

### Graphene and Its Derivatives

**Fig. 3:**
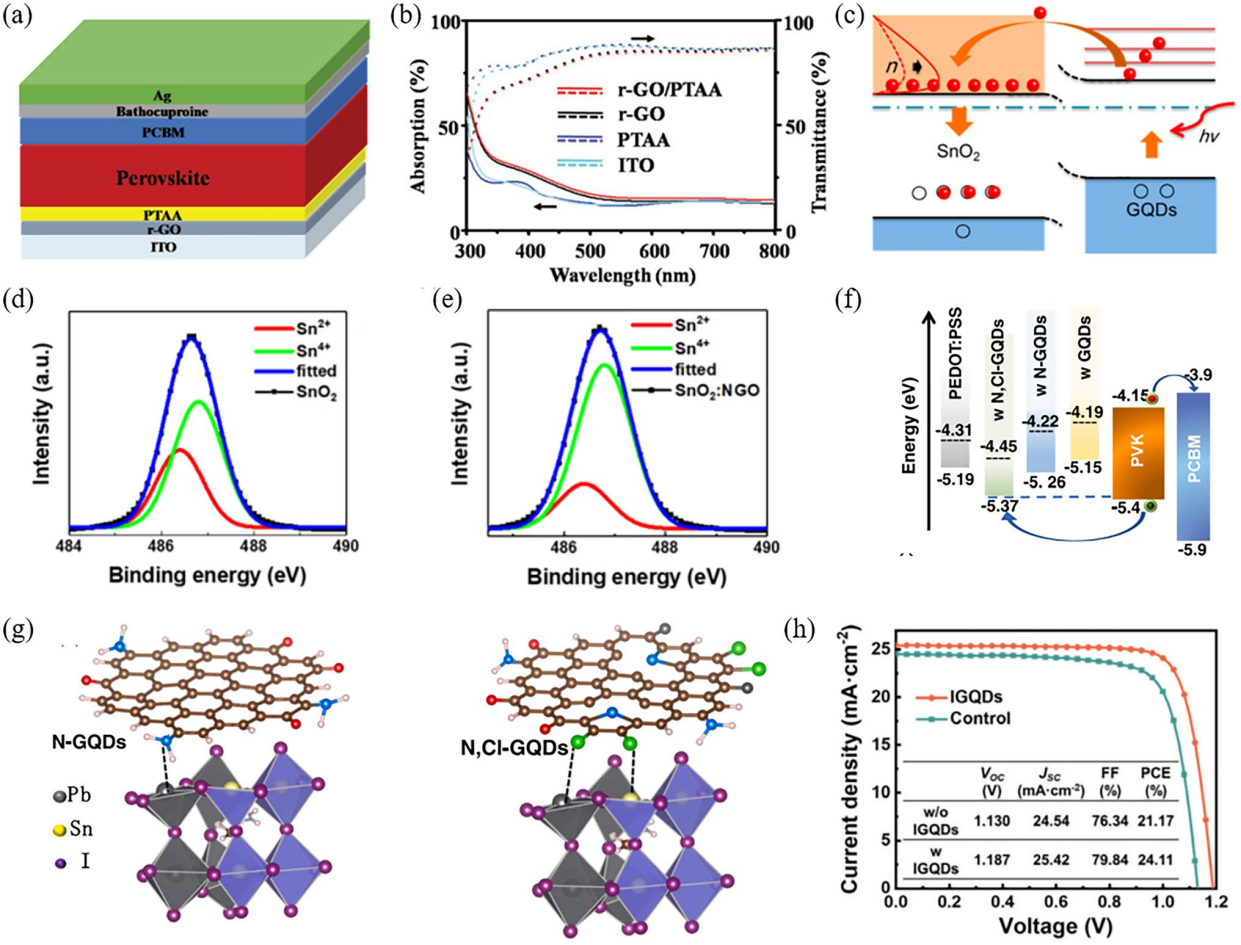
**a** Diagram of the device with a bilayer hole transport system of rGO/PTAA. **b** The absorption and transmission spectra of ITO, PTAA, rGO, and rGO/PTAA. Reproduced with permission from Ref. [78]. Copyright 2017, Wiley. **c** Schematic illustration of the process of hot electron transfer from GQDs to SnO_2_ under illumination. Reproduced with permission from Ref. [134]. Copyright 2017, American Chemical Society. **d, e** X-Ray Photoelectron Spectroscopy of the Sn 3d_5/2_ peak acquired from **d** the pristine SnO_2_ layer and **e** the composite layer of SnO_2_:NGO. Reproduced with permission from Ref. [133]. Copyright 2020, American Chemical Society. **f** Energy level diagram of device components. **g** Schematic illustration of N passivation for under-coordinated Pb and Cl passivation for under-coordinated Pb/Sn in the perovskite. Reproduced with permission from Ref. [124]. Copyright 2022, Elsevier. **h** Current–voltage (*J*-*V*) curves of the devices with and without I-GQDs. Reproduced with permission from Ref. [123]. Copyright 2023, Wiley

Graphene was the earliest discovered and utilized 2D material in PSCs [46]. Due to its high conductivity and carrier mobility, the interface modification by graphene can greatly improve the electrical contact between CTLs and perovskite films [59, 74]. Moreover, the inert and atomically smooth surface of graphene renders it an ideal template for the growth of perovskite materials. For instance, the vdW epitaxial growth of CsPbBr_3_ thin films on a TiO_2_ substrate can be achieved by overlaying a low-defect, large-area monolayer of graphene. This approach enables the formation of a high-quality CsPbBr_3_ film, with an average grain size increased from 0.76 to 1.22 μm and improved (100) orientation [110]. However, the lack of a bandgap in graphene restricts its semiconductor applications. As derivatives of graphene, GO and rGO possess enhanced optoelectronic properties and solvent dispersibility, making them popular choices for applications in PSCs [77]. For example, to address the issue of photostability in poly[bis(4-phenyl)(2,4,6-trimethylphenyl)amine] (PTAA)-based inverted PSCs, a composite hole transport layer (HTL) combining rGO and PTAA has been proposed (Fig. 3a) [78]. When compared to pristine PTAA, the rGO/PTAA exhibits a superior absorptivity in the shortwave bands, thereby impeding the penetration of ultraviolet (UV) light into the perovskite layer (Fig. 3b). Moreover, rGO boasts a valence band maximum (VBM) of -4.97 eV, which forms a graded energy level between PTAA (-5.22 eV) and ITO (-4.7 eV). This strategy enables the PSC to achieve a satisfying PCE of 17.2% (active area of 1.02 cm^2^), with an improved short-circuit current density (*J*_*SC*_) and fill factor (FF) from 19.4 mA cm^−2^ and 69.9% to 20.3 mA cm^−2^ and 77.7%, respectively. Furthermore, the bilayer constructed PSCs exhibit outstanding light-soaking stability, maintaining ~ 90% of its original PCE after continuous illumination for 500 h at 100 mW cm^−2^. As for the regular PSCs, SnO_2_ ETL has been established for improving PCE and stability [131]. However, the semiconductor characteristics of this material strongly rely upon its inherent oxygen vacancies. It is important to maintain appropriate balance in the oxidation state of Sn, as SnO and SnO_2_ exhibit p-type and n-type properties, respectively [132]. By incorporating a modest quantity of nitrogen-doped GO (NGO) as an oxidizing agent into the precursor solution of SnO_2_, the oxygen vacancies are partially passivated and a conspicuous decrement in Sn^2+^ and increment in Sn^4+^ can be observed (Fig. 3d, e) [133]. Due to the enhancement in charge extraction and the reduction of non-radiative recombination, the open-circuit voltage (*V*_OC_) of the device incorporating the SnO_2_: NGO composite layer is 60 meV higher than that of the device with pristine SnO_2_.

By virtue of the intriguing quantum confinement effects, graphene quantum dots (GQDs) exhibit size-dependent bandgaps and remarkable surface-to-volume ratios [135]. These distinguishing features set them apart from conventional 2D graphene and broaden their functionalities at the bottom interface of PSCs. For example, the introduction of small amounts of GQDs with diameters ranging from 5–10 nm into an ethanol solution of SnCl_2_·2H_2_O yields a refined ETL of SnO_2_: GQDs [134]. The optical bandgap of GQDs with such dimensions is ~ 2.4 eV, which is considerably narrower in comparison to that of SnO_2_. In addition, the conduction band minimum (CBM) of GQDs is higher than that of SnO_2_. Therefore, the photo-induced electrons in GQDs spontaneously transfer to the conduction band of SnO_2_, subsequently filling the electron trap-state defects of SnO_2_ (Fig. 3c). As a result, the electron trap-state density and WF of SnO_2_:GQDs decline from an initial value of 4.30 × 10^16^ cm^−3^ and 4.35 eV to 1.23 × 10^16^ cm^−3^ and 4.01 eV, respectively. This modification improved the PCE of the device from the original 17.91% (with *V*_OC_ = 1.101 V, *J*_SC_ = 22.10 mA cm^−2^, FF = 73.6%) to 20.31% (with *V*_OC_ = 1.134 V, *J*_SC_ = 23.05 mA cm^−2^, FF = 77.8%).

In order to further improve the electronic structure and surface properties of GQDs, hetero-element doping and surface functionalization are important approaches [59]. To compare the effects of different doping elements on GQDs, the researchers synthesized three types of quantum dots: pristine GQDs, N-doped GQDs (NGQDs), and N and Cl co-doped GQDs (N,Cl-GQDs) [124]. Subsequently, these quantum dots are used to modulate the interface between (3,4-ethylenedioxythiophene):poly(styrenesulfonate) (PEDOT:PSS) HTL and Sn–Pb mixed perovskite. It has been found that the doping of N and Cl reinforces the p-type characteristics of GQDs and passivates the under-coordinated Sn/Pb cations in Sn–Pb mixed perovskite (Fig. 3f, g). Besides, the π-conjugated effect of the graphene structure and the electronegativity of Cl regulate the charge distribution at the interface, thereby facilitating hole extraction and conduction. The optimized PCE achieved for the three different types of PSCs were 18.6% for GQDs, 20.2% for NGQDs, and a remarkable 21.5% for N,Cl-GQDs. Significantly, the N,Cl-GQDs PSC demonstrated outstanding *V*_OC_ and FF values of 0.886 V and 80.4%, respectively, which are recognized as among the highest reported for Sn–Pb mixed PSCs. Moreover, the introduction of imidazole bromide functionalized GQDs (I-GQDs) at the SnO_2_/CH_4_N_2_HPbI_3_ (FAPbI_3_) interface elevates the carrier mobility of SnO_2_ by a factor of 1.7 and improves the crystal quality of FAPbI_3_. As a result, the PCE of the I-GQDs PSCs increased from 19.57% (with *V*_OC_ = 1.031 V, *J*_SC_ = 24.34 mA cm^−2^, FF = 78%) to 22.37% (with *V*_OC_ = 1.073 V, *J*_SC_ = 25.42 mA cm^−2^, FF = 82%) [122]. In recent progress, the down-conversion characteristics of I-GQDs have been demonstrated. By converting photons in the UV band into long-wavelength photons that can be absorbed, the PSCs incorporating I-GQDs achieved an impressive PCE of 24.11% (Fig. 3h) [123]. After continuous UV irradiation (365 nm, 20 mW cm^−2^) for 300 h, ~ 81% of the initial PCE is retained.

### Transitional Metal Dichalcogenides

**Fig. 4:**
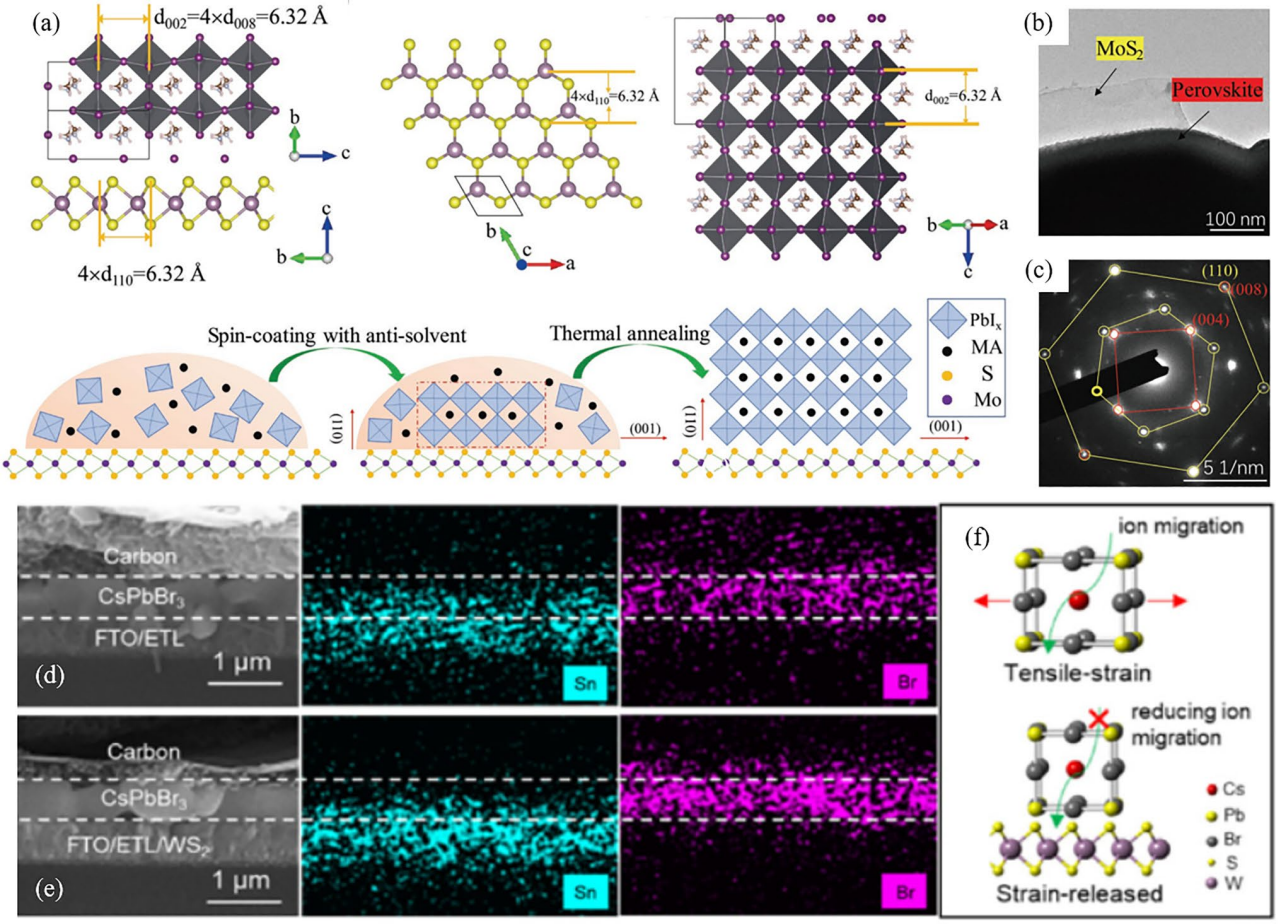
**a** Schematic illustration of the vdW epitaxial growth of a MAPbI_3_ grain on a MoS_2_ surface. The interplanar distances of the MoS_2_ (110) and MAPbI_3_ (008) planes are identical, which is perfect for epitaxial growth. **b** Top-view transmission electron microscopy image of MoS_2_ with a MAPbI_3_ perovskite layer grown on its surface. **c** The selected-area electron diffraction pattern of an overlapping area of MAPbI_3_ and MoS_2_. Two separate diffraction spots are observed: the yellow one belongs to MoS_2_, while the red one belongs to MAPbI_3_. Reproduced with permission from Ref. [111]. Copyright 2019, Wiley. **d, e** Cross-sectional scanning electron microscopy images and their corresponding energy dispersive spectroscopy mapping images of CsPbBr_3_ PSCs **d** without and **e** with WS_2_ modification after aging treatment. **f** Schematic illustration of ion migration in tensile strain- and strain-released perovskite unit cells. Reproduced with permission from Ref. [51]. Copyright 2020, Wiley

As a highly promising 2D material, MoS_2_ displays exceptional carrier mobilities and possesses an energy band structure that is ideally suited for a diverse range of optoelectronic applications [44, 89]. The VBM of the MoS_2_ thin film is approximately -5.41 eV, which closely aligns with that of CH_3_NH_3_PbI_3_ (MAPbI_3_, -5.43 eV) [136]. The energy level alignment indicates MoS_2_ flakes is a promising candidate of interlayers in MAPbI_3_-based PSCs. For example, by adding a layer of MoS_2_ between PTAA and MAPbI_3_, the aging pathways at the interface were effectively suppressed. This approach is advantageous for the preparation of large-area PSCs. For a device with an active area of 0.5 cm^2^, its PCE has been increased from 10.64% (control) to 13.17% (with MoS_2_), with an improved *V*_OC_ from 0.949 to 1.009 V [137]. Furthermore, the MoS_2_-based PSCs achieved a *T*_80_ lifetime of 568 h, which represent the state-of-the-art for PSCs at that time. Due to its smooth and dangling bond-free surface, MoS_2_ also serves as an epitaxial growth template for MAPbI_3_ and form a vdW heterojunction between them. In detail, the MAPbI_3_ (008) and MoS_2_ (110) planes have an identical interplanar distances of 1.58 Å, which promotes the out-of-plane growth of perovskite films with a preferred crystal orientation along the (110) axis (Fig. 4a-c) [111]. As a result, the average grain size of MAPbI_3_ increased from 290 to 526 nm, accompanied by a decrease in defect state density from 2.59 × 10^16^ to 1.17 × 10^16^ cm^−3^. Through the modification of MoS_2_, the PCE of the inverted MAPbI_3_-based PSC increased from the initial 18.12% to 20.55%, with an improved *V*_OC_ from 1.08 to 1.12 V.

On the other hand, the bottom interface undergoes severe compressive or tensile strain during the thermal annealing process, as the thermal expansion coefficient of perovskite is approximately ten times higher than that of the underlying metal oxide layer [138–140]. This residual stress not only hampers carrier transport but also accelerates the aging process of the device. In particular, the all-inorganic CsPbBr_3_ material exhibits a phase transform temperature of up to 250 °C, which is higher than its traditional hybrid perovskite counterpart [141]. This notable difference in temperature gradient is expected to result in an increased tensile strain at the bottom interface. To mitigate this problem, a vdW heterostructure composed of WS_2_/CsPbBr_3_ has been demonstrated [51]. In this situation, WS_2_ acts as a lubricant between CsPbBr_3_ and the metal oxide substrate, thereby alleviating the constraints imposed by the substrate on the expansion and contraction of the perovskite lattice. The cross-sectional energy-dispersive spectroscopy mapping images demonstrate that the migration of ions at interfaces is significantly suppressed due to enhanced interface contact and improved perovskite lattice structure (Fig. 4d-f). Moreover, the average decay time of electrons decreased from 0.575 ns (without WS_2_) to 0.247 ns (with WS_2_). As a result, the PCE of all-inorganic PSCs with an FTO/SnO_2_-TiO_x_Cl_4-2x_/WS_2_/CsPbBr_3_/carbon structure has reached 10.65%, with *V*_OC_, *J*_SC_, and FF of 1.70 V, 7.95 mA cm^−2^, and 79%, respectively. As a comparison, the PCE of the pristine device is 9.27%, with *V*_OC_, *J*_SC_, and FF of 1.59 V, 7.45 mA cm^−2^, and 78.3%, respectively. Moreover, WS_2_ has been employed to modify the interface between the PTAA and the ternary cation perovskite (Cs_0.05_MA_0.05_FA_0.9_PbI_2.7_Br_0.3_) [118]. Here, the researchers prepared WS_2_ nanosheets with an average lateral size of 700 nm and a thickness ranging from 2–5 nm. These nanosheets demonstrated an optical bandgap of 1.76 eV, with a VBM of −5.12 eV and a CBM of −3.36 eV. These energy levels are well-aligned with the energy levels between PTAA and the perovskite film. In addition, the lattice distance on the (020) plane of the Cs_0.05_MA_0.05_FA_0.9_PbI_2.7_Br_0.3_ is 0.31 nm, which is twice that of the (110) plane of WS_2_. This characteristic enhances the compatibility between the two materials and promotes the vdW epitaxial growth of Cs_0.05_MA_0.05_FA_0.9_PbI_2.7_Br_0.3_ atop WS_2_. As a result, the defect state density of the perovskite film was reduced from 1.94 × 10^15^ to 0.68 × 10^15^ cm^−3^. With the remarkable enhancement in *V*_OC_ from 1.08 to 1.15 V, the WS_2_-based PSC achieved a PCE of 21.1%, which is one of the highest reported PCEs for inverted PSCs at that time. For Sn-based perovskites (FASnI_3_), the utilization of MoS_2_, WS_2_, and WSe_2_ as templates has been demonstrated to effectively promote vdW epitaxial growth and produce larger crystalline grains. However, among the three templates, WSe_2_ exhibits the highest VBM, which aligns more favorably with the VBM of FASnI_3_. Consequently, photovoltaic devices incorporating WSe_2_ templates yield the highest photovoltaic performance.

Expect for the application in inverted PSCs, metal sulfides can also be utilized in the bottom interface of regular PSCs. For example, SnS_2_ is a typical binary 2D material with n-type semiconductor properties [142, 143]. It has a bandgap of ~ 2.5 eV, with VBM and CBM of −6.54 and −4.24 eV, respectively. Compared to SnO_2_ ETL, 2D SnS_2_ film displays a superior electron mobility (7.85 × 10^−4^ vs 9.78 × 10^−5^ cm^2^ V^−1^ s^−1^), a higher conductivity (7.17 × 10^−4^ vs 1.78 × 10^−5^ S cm^−1^), and a lower root mean square roughness (RMS, 0.31 vs 2.4 nm). By replacing SnO_2_ with SnS_2_ as the ETL, the loss caused by the imbalance of charge carriers at the ETL/perovskite and perovskite/HTL interfaces is significantly reduced. Due to the enhancements in *V*_OC_ (1.095 to 1.161 V) and *J*_SC_ (22.60 to 23.55 mA cm^−2^), the PCE of SnS_2_-based PSCs improved from 17.72% to 20.12% [144]. This achievement establishes the highest PCE for PSCs using SnS_2_ as the ETL. In a recent breakthrough, an all-in-one SnO_2_-SnS_2_-CsPbBr_3_ interface, with architecture of SnS_2_ [0.316 nm for (100)]/SnO_2_ [0.335 nm for (110)] and SnS_2_ [0.589 nm for (001)]/CsPbBr_3_ [0.587 nm for (001)] directions, was established [112]. Here, SnS_2_ functions as a "bridge" between the incompatible interface of SnO_2_ ETL and CsPbBr_3_ active layer. This exquisite design diminishes the interface barrier, thereby minimizing energy loss as charges traverse the interface. Besides, due to the matched lattice and high-quality epitaxial growth, the defect density of CsPbBr_3_ thin films decreased from 1.80 × 10^16^ to 1.41 × 10^16^ cm^−3^. Through this all-in-one strategy, the all-inorganic, carbon electrode-based CsPbBr_3_ PSC exhibits a boosted PCE of 10.72%, along with an enhanced *V*_OC_ of 1.635 V. After being stored in air at 80% relative humidity (RH) and 25 °C for 700 h, the unencapsulated device retains 90% of its initial PCE.

### MXenes

**Fig. 5:**
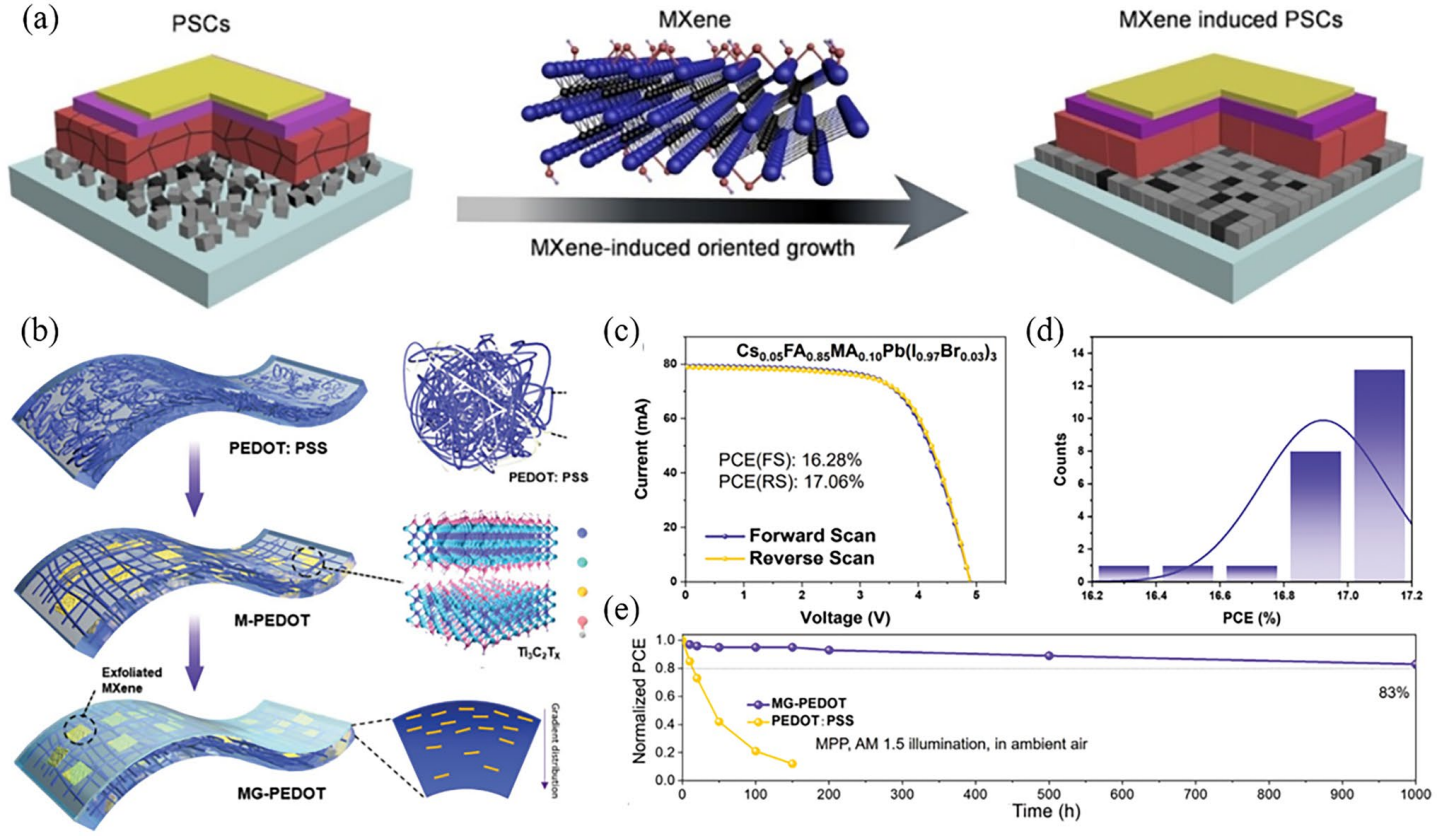
**a** Structural model of the vertical growth of perovskite induced with MXene. Reproduced with permission from Ref. [125]. Copyright 2022, Wiley. **b** Schematic illustration of the nanocomposite MG-PEDOT film preparation. **c-e** Photovoltaic performance of flexible modules based on MG-PEDOT. **c** *J*–*V* curves. **d** Efficiency statistics of 24 separate perovskite solar modules. **e** The stability of encapsulated devices under operational conditions (AM 1.5 illumination) in ambient air. Reproduced with permission from Ref. [126]. Copyright 2023, Wiley

The MXenes, a rapidly expanding family of 2D materials, are well-known for their unique optoelectronic properties and adjustable surface termination [94, 145]. Ti_3_C_2_T_x_ is the first discovered MXenes, which exhibits metallic conductivity, flexibility, hydrophilicity, and an oxide-like surface termination [146]. Notably, UV-ozone treatment is an important approach to enhance the electronic properties of Ti_3_C_2_T_x_. For a regular device utilizing Ti_3_C_2_T_x_ as the ETL, the initial performance was characterized by a modest PCE of 5.00%, with a *V*_OC_ of 0.8 V, *J*_SC_ of 15.87 mA cm^−2^, and FF of 40%. Interestingly, after 30 min of UV-ozone treatment on Ti_3_C_2_T_x_, the PCE substantially improved to 17.17%, with an enhanced *V*_OC_ of 1.08 V, *J*_SC_ of 22.63 mA cm^−2^, and FF of 70% [147]. The X-ray photoelectron spectroscopy indicates that UV-ozone treatment increases the Ti–O bonds on the surface of Ti_3_C_2_T_x_, thereby reducing the non-radiative recombination losses at the Ti_3_C_2_T_x_/perovskite interface. Recently, research has shown that Ti_3_C_2_T_x_ can be used as an additive or passivation layer to improve the interfacial contact between SnO_2_ and the perovskite layer. For example, by doping a small amount of Ti_3_C_2_T_x_ into SnO_2_, the vdW interaction between SnO_2_ nanocrystals is weakened. This refinement leads to an optimized ETL exhibiting an impressive boost in conductivity from 9.62 × 10^–5^ to 1.85 × 10^–4^ S cm^−1^ [125]. In addition, the as-prepared SnO_2_-Ti_3_C_2_T_x_ composite layer promotes the vertical crystal growth of perovskite, with the average grain size increasing from ~ 356 nm to 1 μm (Fig. 5a). As a result, the PCE of the SnO_2_-Ti_3_C_2_T_x_ device enhanced from the initial 20.03%, with *V*_OC_, *J*_SC_, FF of 1.09 V, 24.16 mA cm^−2^, 75.8%, respectively, to 23.07%, with *V*_OC_, *J*_SC_, FF of 1.13 V, 25.07 mA cm^−2^, 81.1%, respectively. In a similar device, by introducing Ti_3_C_2_Cl_x_ as a passivation layer between the SnO_2_ and perovskite layer, the device achieved a high PCE of 24.86%, with *V*_OC_ increasing from 1.173 to 1.203 V and FF rising from 78.05% to 82.14% [127]. This improvement is mainly attributed to the anchoring of Pb^2+^ by Cl_x_ terminals. Despite its excellent performance, Ti_3_C_2_T_x_ is prone to agglomeration, which limits its application in large-area PSCs. In order to address this challenge, a unique MXene/Glucose/PEDOT:PSS nanocomposite HTL has been proposed for flexible large-area perovskite solar module [126]. The incorporation of glucose and its half-caramelization process facilitate the spontaneous exfoliation and redistribution of aggregated Ti_3_C_2_T_x_ MXene nanosheets within PEDOT:PSS (Fig. 5b). Based on this strategy, the flexible perovskite solar minimodule (15 cm^2^) achieved a high PCE of 17.06% and demonstrated excellent repeatability (Fig. 5c–d). After 1000 h of continuous 1-sun illumination, their initial PCE is retained at 83% while performing maximum power point tracking (Fig. 5e).

In comparison to Ti_3_C_2_T_x_, Nb_2_CT_x_ has a larger surface area, which can be attributed to the absence of one atomic layer [148]. This advantage enhances the contact area with perovskite, thereby facilitating the extraction and transport of carriers. Additionally, the plentiful surface groups, including -OH, -O, -F, and -NH_2_, can be readily integrated into Nb_2_CT_x_ MXene [93]. Theoretical calculations have shown that the WF of O-terminated Nb_2_CT_x_ MXene is higher than that of OH-terminated counterparts [149]. The researchers conducted a treatment of Nb_2_CT_x_ with O plasma, which led to an observed increase in its WF from 4.7 to 5.04 eV. Using this as the HTL in an inverted PSC resulted in an enhancement of the PCE from 18.08% to 20.74% [150]. Conversely, when hydrazine (N_2_H_4_) is used, it replaces the surface -F groups with -NH_2_ groups, resulting in a reduction of the WF of Nb_2_CT_x_ from 4.65 to 4.32 eV. Furthermore, the existence of a dipole moment from the Nb layer to the -NH_2_ groups leads to an upward shift of the VBM of Nb_2_CT_x_. This adjustment allows the VBM of Nb_2_CT_x_ to align with that of the perovskite layer. As a result, a considerable PCE of 21.79% has been achieved in a regular PSC that employs Nb_2_CT_x_ as the ETL [128]. Besides, the corresponding flexible and large-area (active area of 0.99 cm^2^) counterparts achieve PCEs of 19.15% and 18.31%, respectively. Due to the inhibition of I^−^ migration by -NH_2_ groups, this type of PSC demonstrates excellent stability. The unencapsulated device retains 93% of its initial PCE after being stored for 1,500 h inside a glovebox.

### Black Phosphorus and Other 2D Materials

As an emerging 2D material, BP possesses a tunable direct bandgap and ambipolar conductivity characteristics [151, 152]. In its polycrystalline form, BP demonstrates impressive electron and hole mobility, with levels as high as 220 and 350 cm^2^ V^−1^ s^−1^, respectively [153]. It is worth noting that the electron mobility of traditional TiO_2_ is only 0.1–4 cm^2^ V^−1^ s^−1^, which is approximately three orders of magnitude lower than that of BP [154]. Therefore, replacing TiO_2_ with BP as the ETL in PSCs is a logical progression. This approach becomes even more advantageous with the promising potential of BP in low-temperature solution-based synthesis, especially for the production of flexible PSCs. For example, through the implementation of the liquid-phase exfoliation technique, the researchers synthesized BP quantum dots (BPQDs) with a diameter distribution ranging from 3 to 10 nm. These BPQDs were then utilized as the ETL in a flexible PSC. After the optimization of the number of BPQD layers (5-layers), the device PEC increased from 3.58% (without BPQDs) to an impressive 11.26% (with BPQDs) [155]. Moreover, recent research revealed that the incorporation of a small amount of BPQDs in CsFAMA perovskite can raise its energy barrier for defect formation, which is supported by the observed reduction in defect state density from 2.83 × 10^16^ to 8.96 × 10^15^ cm^−3^. Benefitting from it, the PCE of the PSC incorporating of BPQDs improved from 19.13% to 22.85% as its *V*_OC_ was enhanced from 1.17 to 1.22 V [156]. Notably, the *V*_OC_ ranks among the highest values for perovskite film with a bandgap of ~ 1.60 eV.

**Fig. 6:**
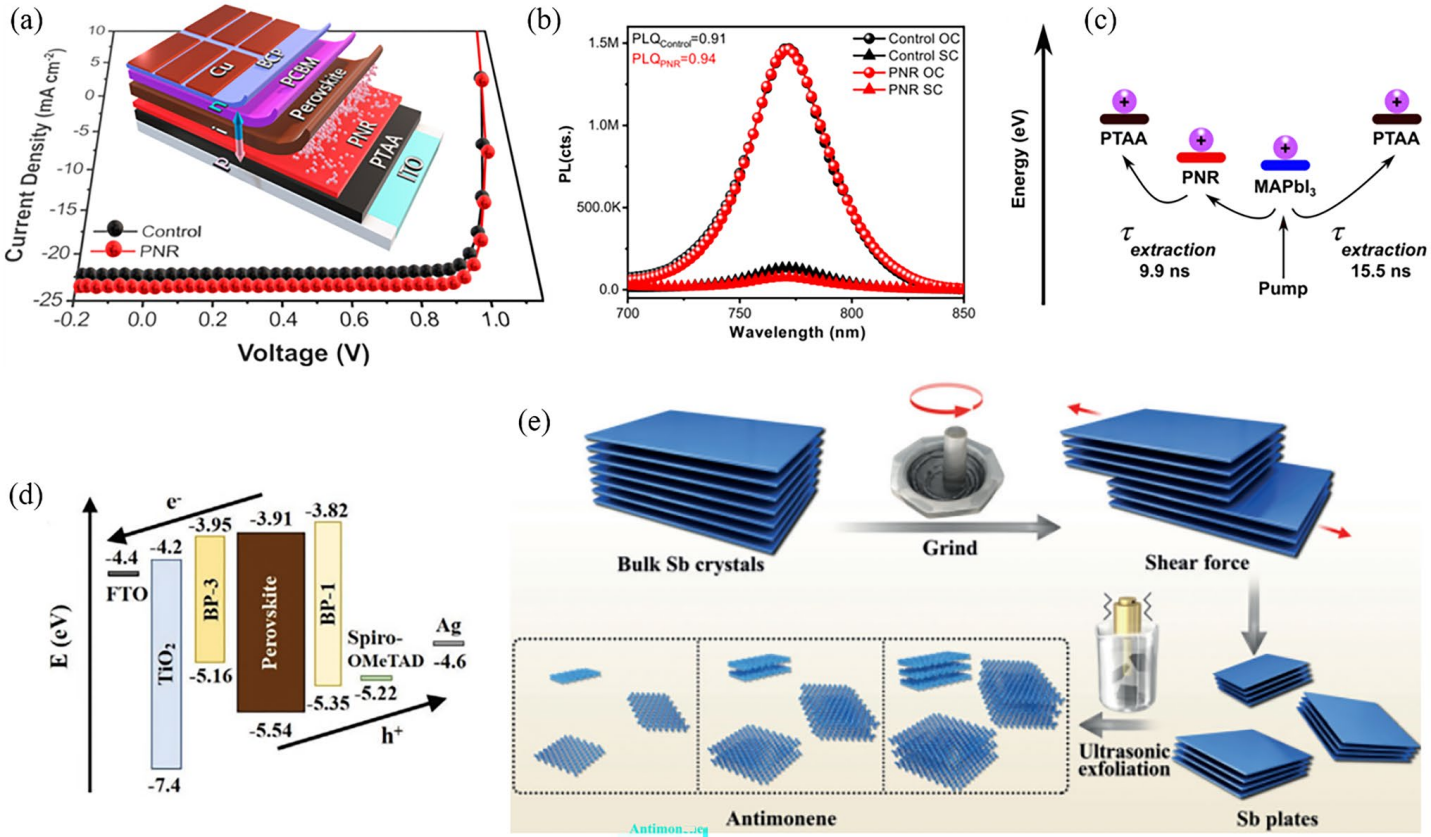
**a** Schematic diagram of PNRs-modified inverted PSC and the corresponding *J*-*V* curves. **b** Photoluminescence of control and PNRs devices under open-circuit and short-circuit conditions. **c** Proposed diagram of enhanced hole extraction facilitated by PNRs. Reproduced with permission from Ref. [130]. Copyright 2021, American Chemical Society. **d** Energy level diagram of each constituent in PSC with dual-positioning of BP at both interfaces. Reproduced with permission from Ref. [129]. Copyright 2020, Wiley. **e** Schematic illustration of the fabrication process for antimonene. Reproduced with permission from Ref. [157]. Copyright 2018, Wiley

Besides the utilization as an ETL, the bipolar transport nature of BP also makes it a popular option for modifying the HTL. For instance, remarkable enhancements in performance have been achieved by incorporating phosphorene nanoribbons (PNRs) as charge-selective interlayers at the PTAA/MAPbI_3_ interface (Fig. 6a) [130]. The hole extraction time was reduced from 15.5 to 9.9 ns, while the conductivity of HTL witnessed an enhancement from 9.25 × 10^−7^ to 1.02 × 10^−6^ S cm^−1^ (Fig. 6b, c). This modulation has resulted in a high FF of 83% for the corresponding inverted PSCs, with an PCE exceeding 21%. Moreover, it has been found that the bandgap and conduction structure of BP can be facilely tuned by manipulating the spin-coating times of BP dispersion on the substrates [129]. Building upon this understanding, researchers designed an device that incorporates a cascade conduction band at both the ETL/perovskite and perovskite/HTL interfaces (Fig. 6d). This innovative approach improved charge extraction and boosted the overall performance of the device.

Besides, other 2D materials such as antimonene, h-BN, CNs, and MOFs have been reported for the modification of the bottom interface of PSCs [67, 68, 158, 159]. Among them, antimonene seems to have great application prospects. Antimonene is a 2D material similar to BP in terms of properties, while demonstrates exceptional stability in both its physical and chemical characteristics [99]. Theoretical calculations indicate that as antimonene transitions from bulk to monolayer, its material properties undergo a transformation from semimetallic to semiconducting. Throughout this process, the band gap of antimonene increases from 0 to ~ 2.28 eV [101]. Furthermore, the semiconducting form of antimonene exhibits high carrier mobility, exceptional thermal conductivity, and spin-electronic properties [160, 161]. However, the production of high-quality, large-area thin antimonene layers has been challenging in experiment due to the short interlayer distance and high binding energy of antimonene. In order to address this issue, a method has been developed that includes a pre-grinding step, followed by liquid-phase exfoliation aided by sonication (Fig. 6e) [157]. By subjecting the mortar to pre-grinding, a shear force is applied along the surfaces of the layers. This process facilitates the formation of large, thin antimony plates, which can be conveniently exfoliated into smooth and sizable antimonene sheets. When the as-prepared antimonene was utilized as the HTL in PSCs, the *J*_SC_ of the corresponding devices increased from 11.2 (in the absence of an HTL) to 14.6 mA cm^−2^. Encouragingly, the incorporation of a monolayer of antimonene between PTAA and MAPbI_3_ yielded an augmentation in *J*_SC_ from 21.69 to 23.52 mA cm^−2^ [67]. These findings confirm the rapid extraction and efficient hole transportation abilities of antimonene, establishing it as a promising candidate for future high-performance PSCs.

## 2D Materials at Top Interfaces

The top interface refers to the interface between the perovskite layer and the back electrode. For a regular device, the top interface includes the interfaces of perovskite/HTL and HTL/back electrode. Therefore, the top interface extracts photogenerated holes from the active layer while obstructing the backflow of electrons [25, 26]. On the other hand, the top interfaces of inverted device have an opposite configuration and direction of charge transport. Unlike the bottom interface, the top interface does not exert a direct influence on the light absorption and film quality of the perovskite layer. However, the rapid crystallization and ionic nature of perovskite materials give rise to numerous defects on its surface, including dangling bonds, voids, and free charges, which limit the efficiency and stability of the PSCs [30, 162]. Additionally, due to the lack of isolation from bulk perovskite, the top interface is more vulnerable to external stimuli such as water, oxygen, and heat. Besides, the chemical reactions occurring at the top interface, which involve the migration of halide anions and the diffusion of metal atoms, result in severe degradation of the perovskite materials [36, 48, 50]. Taking into account these considerations, the incorporation of buffer layers at both the interfaces between the perovskite and CTLs, as well as between the CTLs and the back electrode, is an important strategy to enhance the PCE and stability of the PSCs. The hydrophobicity, compactness, and chemical stability of these interfacial materials play a pivotal role in the success of this approach. In Sect. 4, we summarize the utilization of 2D materials at the top interface of PSCs, focusing on their roles as an active buffer layer (ABL) and interlayer. Representative 2D materials at top interface for the enhancement of device performance and stability are illustrated in Table 3.

**Table 3:** Representative 2D materials at the top interface for improving the efficiency and stability of PSCs

2D materials	Device structure	Function	PCE (%)	Characteristic and stability	Refs
Graphene	FTO/SnO_2_/FAPbI_3_/spiro-OMeTAD/EVA/graphene/Cu-Ni/graphene	Inhibit ion migration metal diffusion, energy level alignment	24.34	95% its initial PCE after 5,000 h of tracking at maximum power points under continuous 1-sun illumination	[36]
Oxo-Graphene	ITO/MeO-2PACz/CsFAMA:Oxo-Graphene /C_60_/BCP/Ag	Provide anchoring sites to bind the excess PbI_2_ in perovskite, passivate grain boundaries	23.7	93.8% its initial PCE after 1,000 h of tracking at maximum power points under continuous 1-sun illumination	[163]
rGO	FTO/TiO_2_/CsFAMA/CuSCN/rGO/Au	As a spacer layer, suppress potential-induced degradation of the CuSCN/Au contact	20.4	All-inorganic HTL, > 95% its initial PCE after 1000 h of tracking at maximum power points under continuous 1-sun illumination at 60 °C	[48]
Cl-GO	ITO/SnO_2_/FAMAPbI_3_/Cl-GO/PTAA/Au	Form strong Pb–Cl and Pb–O bonds, impede the loss of decomposed components from soft perovskites	21.08	Active area of 1.02 cm^2^, 90% of its initial PCE for 1,000 h under 1-sun of illumination at 60 °C	[50]
Ti_1_-rGO	FTO/c-TiO_2_/mp-TiO_2_/CsFAMA/spiro-OMeTAD/Ti_1_-rGO/FTO	Improve electrical contact of interface, energy level alignment	21.6	98% and 95% of its initial PCE for 1,300 h under 1-sun of illumination at 25 and 60 °C, respectively	[52]
QD/GO	ITO/SnO_2_/perovskite/(QD/GO)/spiro-OMeTAD/Au	Improve charge transport, suppress ion and moisture diffusion	18.55	Minimodule (36 cm^2^), 90% of its initial PCE for 1,000 h under 1-sun of illumination at 60 °C, in ambient air	[164]
MoS_2_	FTO/c-TiO_2_/SnO_2_/CsFAMA/MoS_2_/PTAA/Au	Suppress interfacial charge accumulation, serve as a complementary layer to the dopant-free HTL	18.54	Dopant-free HTL, 80% of its initial PCE for 45 h under 1-sun of illumination with 45%–50% RH, at room temperature, unencapsulated	[165]
WS_2_	FTO/TiO_2_/CsPbBr_3_/WS_2_/AgIn_5_S_8_/Carbon	Function as HTL, building a convenient pathway for hole transport via Pb–S-W bonds	10.24	All-inorganic, over 93% of its initial PCE after being stored in air with high humidity and temperature (85% RH, 85 °C) for 720 h	[166]
SnS_1−x_O_2x_	ITO/SnO_2_/CsFAMA/(BA)_2_PbI_4_/SnS_1−x_O_2x_/spiro-OMeTAD/Au	Promote carrier transport via Sn-I bonds, enhance the conductivity and hydrophobicity of HTL	24.5	94.5% of its initial PCE after being stored in air with 20%–40% RH for 720 h	[167]
F-BP	FTO/c-TiO_2_/mp-TiO_2_/CsFAMA/F-BP/spiro-OMeTAD/Au	Mitigate the density of trapped states, enhances antioxidant and moisture resistance	22.06	95% of its initial PCE after 30 days storage with 50% RH at room temperature, unencapsulated	[168]
BPQDs	FTO/SnO_2_/CsFAMA/Cs_3_TbCl_6_QDs/spiro-OMeTAD:BPQDs/Au	Improve the hole mobility and conductivity of HTL	23.49	88% of its initial PCE after 2,520 h in ambient air with 30% RH, unencapsulated	[169]
g-C_3_N_4_	PDMS/PEDOT:PSS/FASnI_3_:g-C_3_N_4_/C_60_/BCP/Ag	Delay crystallization, enhance hydrophobicity and oxidation resistance	8.56	91% of its initial PCE after 1,000 h in N_2_ environment; 92% of its initial PCE after 600 cycles at a curvature radius of 3 mm	[69]
Ti_3_C_2_T_x_	ITO/PTAA/CsPbI_3_/Ti_3_C_2_T_x_/CPTA/BCP/Ag	Strengthen the electric field at the perovskite/ETL interface, improve charge separation	19.69	85% of its initial PCE for 1,000 h under 1-sun of illumination with 85% RH and 85 °C	[170]
Nb_2_CT_x_	FTO/SnO_2_/Nb_2_CT_x_/CsFAMA/Nb_2_CT_x_/spiro-OMeTAD/Ag	Energy levels offsets reduction, conduct hole current from grain boundaries to the HTL	24.11	93% its initial PCE after 1,500 h test under an ambient condition with 10%–20% RH	[171]

### Graphene and Its Derivatives

**Fig. 7:**
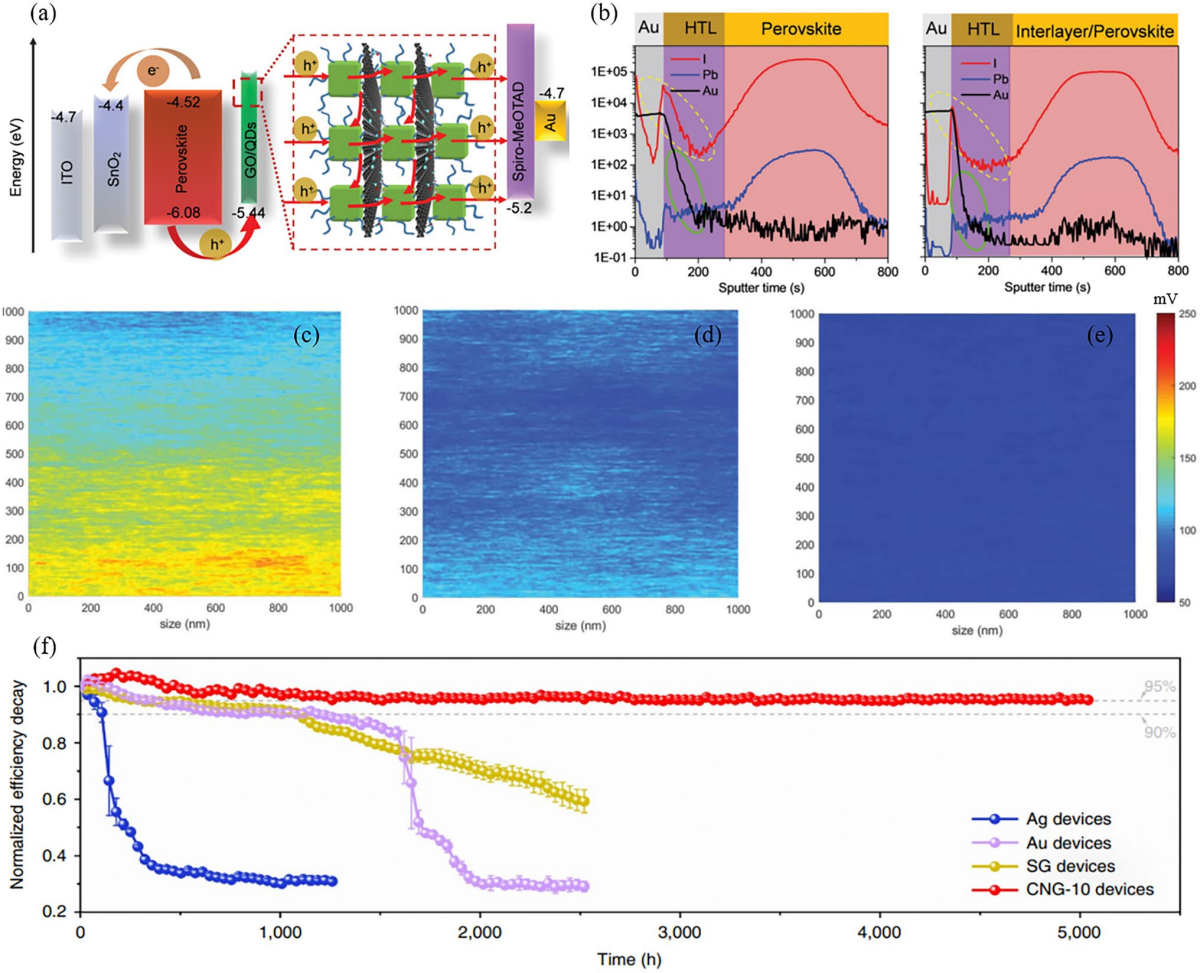
**a** Schematic illustration of the energy levels and charge transport mechanism of PSC with a GO/QDs interlayer. **b** Time-of-flight secondary ion mass spectroscopy depth profiles of iodide ion, Au, and Pb distribution in PSCs without and with the GO/QDs interlayer. Reproduced with permission from Ref. [164]. Copyright 2022, The Royal Society of Chemistry. **c-e** Potential mapping images of **c** perovskite/PTAA, **d** perovskite/GO/PTAA, and **e** perovskite/Cl-GO/PTAA measured by kelvin probe force microscope. Reproduced with permission from Ref. [50]. Copyright 2019, AAAS. **f** The operational stability of PSCs with different electrodes at the maximum power point under one sun illumination. The SG and CNG-10 represent the control sample and the Cu–Ni alloy protected by graphene with 10 layers, respectively. Reproduced with permission from Ref. [36]. Copyright 2022, Springer Nature

In contrast to the bottom interface, the modification of the top interface is considered a post-treatment technique, as it occurs after the crystallization of the perovskite layer has been completed. Due to its remarkable compactness and chemical inertness, the incorporation of graphene and its derivatives as an interlayer material between the perovskite and the back electrode significantly enhances the operational stability of PSCs. For example, researchers replaced the expensive 2,2',7,7'-tetrakis(N,N-p-dimethoxyphenylamino)-9,9'-spirobifluorene (spiro-OMeTAD) with inorganic copper(I) thiocyanate (CuSCN)/rGO to construct devices with a structure of FTO/TiO_2_/CsFAMAPbI_3–x_Br_x_/CuSCN/rGO/Au and achieved comparable PCE with the spiro-OMeTAD-based counterpart (20.8% vs 20.4%) [48]. In this case, rGO acts as an ABL that inhibits the diffusion of ions and metals at the top interface. Combined with the stability of CuSCN, the PSC containing rGO was maintained at 95% of the initial PCE after aging for 1,000 h at the maximum power point under 1-sun illumination and at a temperature of 60 °C. In contrast, the PSC without rGO exceeded 50% of PCE loss. In recent advancements, a composite electrode of copper–nickel (Cu–Ni) has been reported to replace the noble metal electrodes. This alloy is encapsulated with in situ grown bifacial graphene, which improves the stability of the electrode and aligns its energy levels with those of the HTL. The resulting device achieved a high PCE of 24.34% and exhibited 5,000 h operational stability at the maximum power point under continuous 1-sun illumination (Fig. 7f) [36]. Although the favorable performance exhibited by small-area PSCs, they still face challenges when it comes to scaling up from laboratory cells to industrial modules [28, 172]. It has been observed that scalable fabrication can result in increased defects, pinholes, impurities, and deficient contact areas at the interfaces. These detrimental outcomes trigger an amplified leakage current and non-radiative recombination, which compromise the efficiency and long-term stability of the PSCs [173, 174]. To tackle this concern, a strategy of synergistic interface modulation has been proposed by crosslinking CsPbBr_3_ quantum dots (QDs) and conductive GO through a Pb–O bond [164]. The ensuing composite (GO/QDs) show a uniform distribution on the large scan, which offer unparalleled charge transport and efficient interfacial passivation (Fig. 7a). Additionally, the time-of-flight secondary ion mass spectroscopy depth profiles indicate that the GO/QDs interlayer serves as a reliable barrier to hinder the diffusion of ions and metal at the top interface (Fig. 7b). As a result, the PCE of the minimodules (area of 17.11 cm^2^) has witnessed an enhancement from 16.56% to 18.55%. The certified steady-state PCE of this device reaches 17.85%, positioning it among the highest certified efficiencies for minimodule PSCs. After exposure to continuous irradiation of 1-sun for a duration of 1,000 h at 85 °C and 60% RH, the module PCE remains at a remarkable 91%.

In order to make GO more closely combine with perovskite and CTL, doping with heteroatoms such as chlorine and fluorine is essential. For example, by introducing a layer of chlorinated GO (Cl-GO) between perovskite and PTAA, the researchers constructed a perovskite/Cl-GO/HTL heterojunction, and compared it with GO and control (without interlayer) samples [50]. On an aperture area of 1.02 cm^2^, the PCEs of the three devices were 20.00% (control), 20.29% (GO), and 21.08% (Cl-GO), respectively. After operating at the maximum power point for 1,000 h at 60 °C, the PCE of the Cl-GO device remains 90%, whereas the control cell and the cell with GO experienced reductions of 65% and 50%, respectively. This improvement is attributed to the formation of strong Pb-Cl and Pb–O bonds, which reduces the barrier at the top interface (Fig. 7c-e). Furthermore, the use of fluorinated graphene (FG) as an additive in spiro-OMeTAD has been shown to have a versatile impact on improving the performance of PSCs [175]. Firstly, the p-type features of graphene have been strengthened due to the incorporation of highly electronegative fluorine. Secondly, the 2D network structure of FG has the ability to eliminate existing pinholes in spiro-OMeTAD and enhance its hydrophobic properties. This enhancement helps to increase the device resistance to moisture in the air. Finally, the introduction of impurity atoms disrupts the inherent structure of graphene, which leads to an increased number of adsorption sites. As a result, the FG modified PSC achieves a PCE of 23.14%, which surpasses the control device by 11.8%. After a 2,400 h operation under ambient conditions with 25% RH, this graphene-enhanced device retains 90% of its initial PCE.

**Fig. 8:**
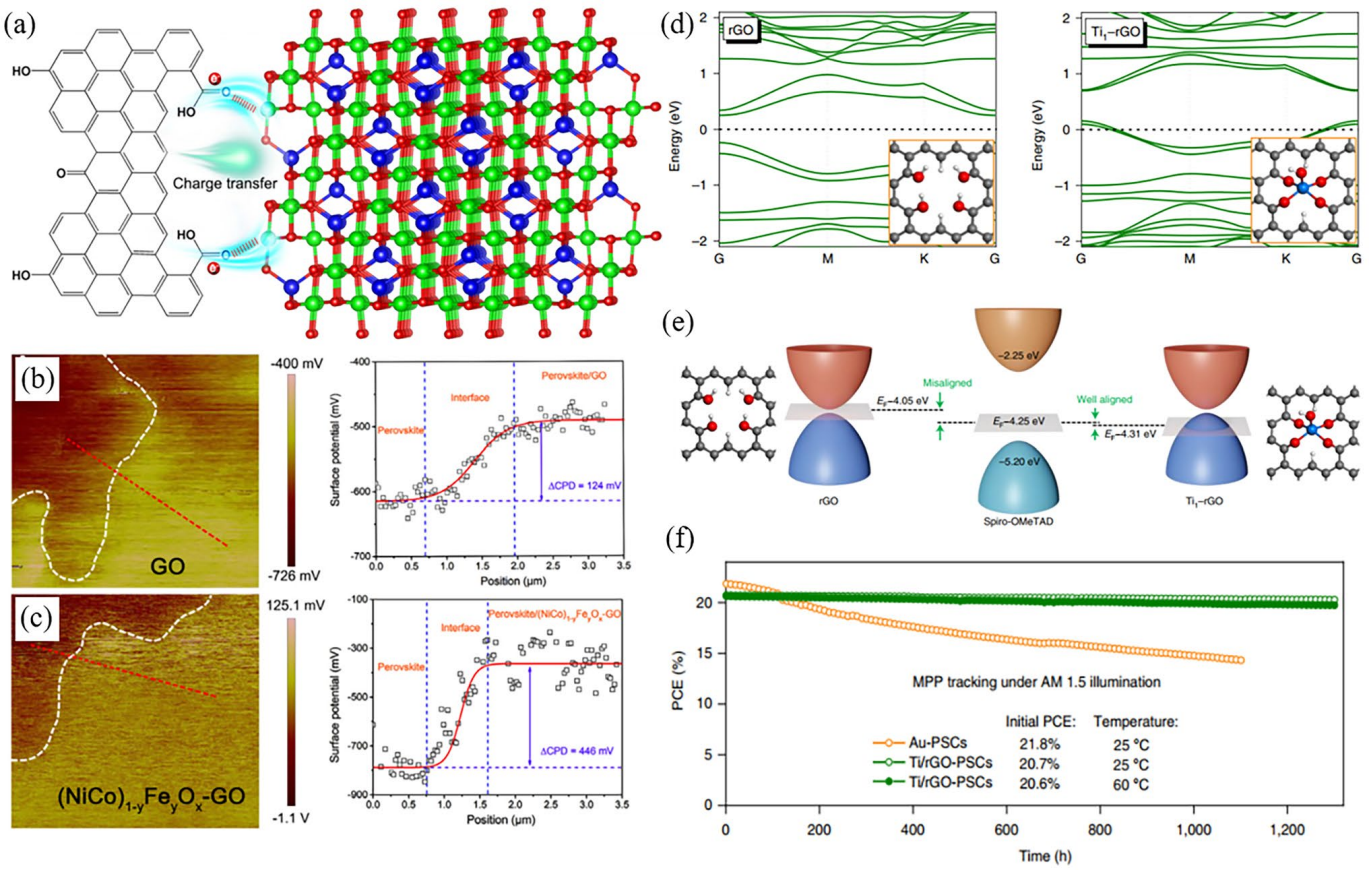
**a** Schematic illustration of the transfer of charge from GO to inorganic NPs. **b, c** Surface potential patterns and corresponding surface contact potential difference values (obtained along the red lines) at the **b** GO/perovskite and **c** NP-GO/perovskite interface. Reproduced with permission from Ref. [177]. Copyright 2021, Wiley. **d** The energy band structure of rGO and Ti_1_–rGO calculated by density functional theory. **e** Schematic band alignment of between spiro-OMeTAD and electrode materials (rGO and Ti_1_–rGO). **f** Maximum power point aging of Ti_1_–rGO-based C-PSC (25 and 60 °C, respectively) and conventional thermal evaporation Au–PSC (25 °C) under an N_2_ atmosphere, one-sun continuous illumination. Reproduced with permission from Ref. [52]. Copyright 2021, Springer Nature

Although GO offers numerous benefits for charge extraction and device protection, it also poses a notable challenge due to the oxygen-containing functional groups on its surface. These groups serve to extract holes but localize charges, leading to a conflict in maximizing both hole extraction and charge transfer [176]. To address this issue, an inorganic nanomaterial, (NiCo)_1-y_Fe_y_O_x_ nanoparticles, has been utilized as a modifier for GO (denoted as NP-GO) [177]. Based on the disparity in electronegativity, electrons from the oxygen-containing groups will spontaneously transfer to the inorganic NPs. This phenomenon results in the p-doped GO and induces surface-oriented dipole motion (Fig. 8a). Consequently, the enhanced hole extraction of GO leads to a reduction in charge recombination loss at the perovskite/HTL interface, which is supported by the surface potential patterns of the top interfaces (Fig. 8b, c). As a result, the all-inorganic PSC fabricated using the structure of FTO/c-TiO_2_/CsPbIBr_2_/NP-GO/carbon has reached a PCE of 10.95%. This represents a notable improvement of 7.38% compared to the reference cell. Due to the protective effect of NP-GO, this device retains 90% of its initial PCE even after being subjected to 70 days of aging in 10% RH air conditions. Recently, a chemisorption method has been employed to introduce a single titanium (Ti) atom onto the surface of rGO, which leads to the formation of a single-atom material (SAM) of Ti_1_-rGO [52]. In this particular case, the Ti adatoms were anchored to the rGO surface through oxygen atoms. By employing density functional theory calculations, noticeable charge transfer between rGO and Ti adatoms has been revealed (Fig. 8d). This phenomenon leads to a decrease in the Fermi level of rGO from -4.05 to -4.31 eV, which is better matched with that of spiro-OMeTAD (-4.25 eV) (Fig. 8e). By incorporating Ti_1_-rGO as the back electrode in carbon-based PSCs (C-PSCs), the series resistance, interfacial contact, and band alignment mismatch between the carbon electrode and adjacent functional layer are minimized. Consequently, the *V*_OC_ and FF of the C-PSCs increased from their initial values of 0.998 V and 25.6 mA cm^−2^ to improved levels of 1.059 V and 26.0 mA cm^−2^, respectively. In line with this, the PCE of the device experiences a significant increase, rising from 17.7% to 21.6%, approaching the PCE of Au-PSCs at 23.5%. Even without encapsulation, the devices demonstrate exceptional stability, with 98% and 95% retention of their initial values after 1,300 h of one-sun illumination at 25 and 60 °C, respectively (Fig. 8f).

### Transitional Metal Dichalcogenides

**Fig. 9:**
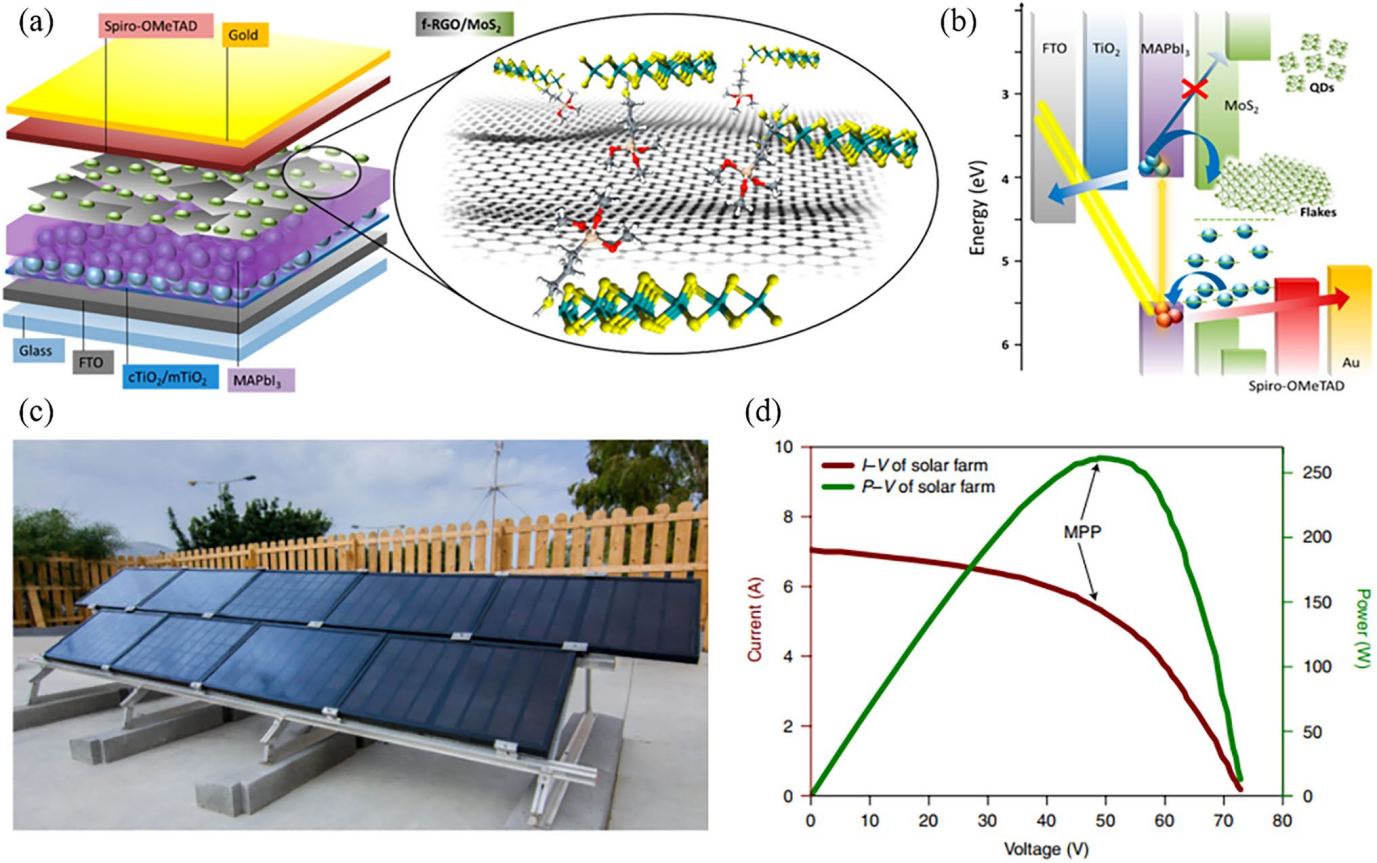
**a** Schematic illustration and **b** energy level diagram of PSC modified with MoS_2_ QDs:f-rGO hybrids. Reproduced with permission from Ref. [183]. Copyright 2018, American Chemical Society. **c, d** Integration of panels into a stand-alone solar farm. **c** Nine panels integrated in a stand-alone solar farm-powered infrastructure installed in Crete, HMU campus. **d** Typical *I–V* and *P − V* characteristics of the solar farm with a maximum output power (P_m_) of ~ 261 W_p_. Reproduced with permission from Ref. [53]. Copyright 2022, Springer Nature

As growth templates, TMDs have found widespread applications at the bottom interface, but reports on their utilization at the top interface are comparatively scarce. This disparity might be attributed to the distinct band structure and conductivity characteristics of these materials. MoS_2_ stands out as a successful example among TMDs, with its combination of high hole mobility and the ability to form favorable heterojunctions with perovskites. Consequently, it is frequently mentioned as a promising HTL or ABL for PSCs. However, the WF of pristine MoS_2_ is below 4.8 eV, which is lower than that of most commonly used HTMs [178]. For example, spiro-OMeTAD has a higher WF of 4.9 eV, while the WF of PEDOT: PSS ranges from 5.0 to 5.2 eV [179–181]. This suggests that 2D MoS_2_ may have insufficient capacity for hole extraction. In addition, the narrow optical bandgap (~ 1.2–1.8 eV) of 2D MoS_2_ positions its CBM at ~ -4.3 eV, which is lower than the lowest occupied energy level of the MAPbI_3_ (~ -4.0 eV) [182]. This characteristic hinders its effectiveness of electrons transport. In order to address this issue, a vdW hybridization architecture composed of zero-dimensional MoS_2_ quantum dots (MoS_2_ QDs) and rGO has been proposed [183]. These two materials are interacted through either the vdW physical adsorption of sulfur-sulfur (S–S) bonds or the S-vacancies passivation/filling (Fig. 9a). As a result of the quantum confinement effect, the optical bandgap and CBM of MoS_2_ is increased from 1.4 eV to over 3.2 eV and from -4.3 to -2.2 eV, respectively, which helps to reduce the backflow of electrons into the HTL (Fig. 9b). On the other hand, the rGO flakes plug the pinholes in the MoS_2_ QDs films, ensuring complete coverage of the perovskite film by this hybridized HTL. The synergy of MoS_2_ QDs and rGO has enhanced the *J*_SC_ (20.28 to 22.81 mA cm^−2^) and *V*_OC_ (1.07 to 1.11 V) of the MAPbI_3_ based PSCs, leading to an increase in the PCE from 16.85% to 20.12%. Similarly, the energy level of MoS_2_ can also be regulated by linking thiol group of 3-mercaptopropionic acid (MPA) moieties on its surface [184]. As an ABL between perovskite and spiro-OMeTAD, this chemically functionalized MoS_2_ (fMoS_2_) exhibited high FF and *J*_SC_ for large-area PSMs. Consequently, the fMoS_2_ decorated PSMs with active areas of 82 and 108 cm^2^ achieved PCE of 15.3% and 13.4%, respectively. As a comparison, the PCE for their pristine MoS_2_ counterparts is 13.56% and 12.5%, respectively. In a recent breakthrough, by modifying the bottom and top interfaces with graphene and fMoS_2_, respectively, the PCE of the perovskite module with aperture area of 101 cm^2^ reached 16.4% [53]. Encouragingly, perovskite panels assembled with the modules reached a peak power exceeding 250 W under outdoor conditions (total area of 4.5 m^2^). As a stand-alone solar farm infrastructure, these panels have been operating for 12 months and achieved a remarkable *T*_*80*_ of 5,832 h (Fig. 9c, d). These results indicate a significant advancement in the practical utilization of perovskite photovoltaic technology.

Besides, it has been discovered that MoS_2_ serves as a complementary layer to the dopant-free HTL due to its fast vertical charge transport and robust chemical stability. For example, in regular PSCs with CsFAMA ternary cations, an ultrathin layer of MoS_2_ is sandwiched between the perovskite and the dopant-free PTAA [165]. The VBM of MoS_2_ is -5.4 eV, which shows an improved energy level alignment and reduces the barrier between PTAA (− 5.1 eV) and CsFAMA (− 5.65 eV). The serial resistance of the modified device decreased from 3.21 to 2.85 Ω cm^−2^, while the shunt resistance increased from 3.85 to 7.09 kΩ cm^−2^. These improvements in electrical contact have enhanced the *V*_OC_ (0.887 to 1.05 V) and FF (74.64% to 79.96%) of PSC, resulting in an increase in PCE from 15.04% to 18.54%. Due to the absence of doping in PTAA and the enhanced hydrophobicity of the interface provided by MoS_2_, this device exhibits strong moisture resistance under ambient conditions. In addition, it has been demonstrated that the incorporation of MoS_2_ into flexible PSCs can enhance both their mechanical and light stability. Through the surface modification of the perovskite with 1-dodecanethiol (DT) and MoS_2_, the polyethylene terephthalate (PET)/ITO-based PSC exhibits complete recovery in terms of PCE, *V*_OC_, *J*_SC_, and FF after undergoing 300 cycles of bending tests [185]. Even after a conventional light exposure cycle, the unencapsulated device manages to retain an impressive 95% of its original PCE. In contrast, the control sample experiences a significant decline, dropping to only 32% of its original PCE under the same conditions.

### MXenes

Given the ability to finely tune the WF of MXenes over a wide range during their synthesis (1.6 eV for OH terminations to 6.25 eV for O terminations), this characteristic enables facile control of the energy levels of perovskite and CTLs [186]. For instance, by co-doping the MAPbI_3_ absorber layer and 1-(3-methoxycarbonyl)-propyl-1-phenyl-(6,6)-C_61_ (PCBM) with Ti_3_C_2_T_x_, it was observed that the WF of the two were adjusted by ~ 60 and ~ 200 meV, respectively [187]. This improvement facilitates the extraction and transport of photogenerated electrons across the top interface. In addition, the facile surface chemical modification of Ti_3_C_2_T_x_ allows for straightforward adjustment of its optoelectronic properties. By incorporating Lewis acid CdCl_2_ during the ultrasonic exfoliation of the Ti_3_AlC_2_, Ti_3_C_2_Cl_x_ terminated with Cl- can been obtained. The as-prepared 2D nanosheets have advantages for use in all-inorganic CsPbBr_3_ PSCs with a configuration of FTO/SnO_2_-TiO_x_Cl_4−2x_/CsPbBr_3_:Ti_3_C_2_Cl_x_/Ti_3_C_2_Cl_x_/carbon [188]. Due to the strong bonding energy of Cl atoms, the Pb^2+^-Cl-Ti_3_C_2_ bridging connection at the top interface functions as a lattice "tape" that restrains the expansion of the perovskite. This mechanism improves the crystalline quality of perovskite films and enhancing the thermal stability of the interfaces. As a result, the Ti_3_C_2_Cl_x_-modified device demonstrates an enhancement in PCE from 9.18% (with *V*_OC_ = 1.569 V, *J*_SC_ = 7.32 mA cm^−2^, FF = 79.9%) to 11.08% (with *V*_OC_ = 1.702 V, *J*_SC_ = 7.87 mA cm^−2^, FF = 82.7%). These achievements place it at the forefront in the field of all-inorganic CsPbBr_3_ PSCs. Similarly, introducing fluorine functionalized MXene QDs (Ti_3_C_2_F_x_ QDs) as a passivation layer between the CsPbI_3_ absorber layer and the sprio-OMeTAD HTL, the device exhibits an excellent PCE of 20.44% with a high *V*_OC_ of 1.22 V [189]. Moreover, in order to suppress the migration of iodide ions from the perovskite layer to the Ag cathode, tetrabutylammonium bromide-modified Ti_3_C_2_T_x_ (TBAB-Ti_3_C_2_T_x_) was developed as a cathode buffer layer (CBL) between PCBM and the Ag cathode [190]. The calculations by density functional theory indicate that partial of the charge in TBAB transfers to Ti_3_C_2_T_x_ via the N and Br atom. This mechanism improves the conductivity of TBAB-Ti_3_C_2_T_x_ and reduces its WF from 4.5 to 3.9 eV, which is more compatible with the energy level of MAPbI_3_. The PCE of the TBAB-Ti_3_C_2_T_x_-based device reached 21.65%, exceeding that of the device with BCP as the CBL (19.94%).

**Fig. 10:**
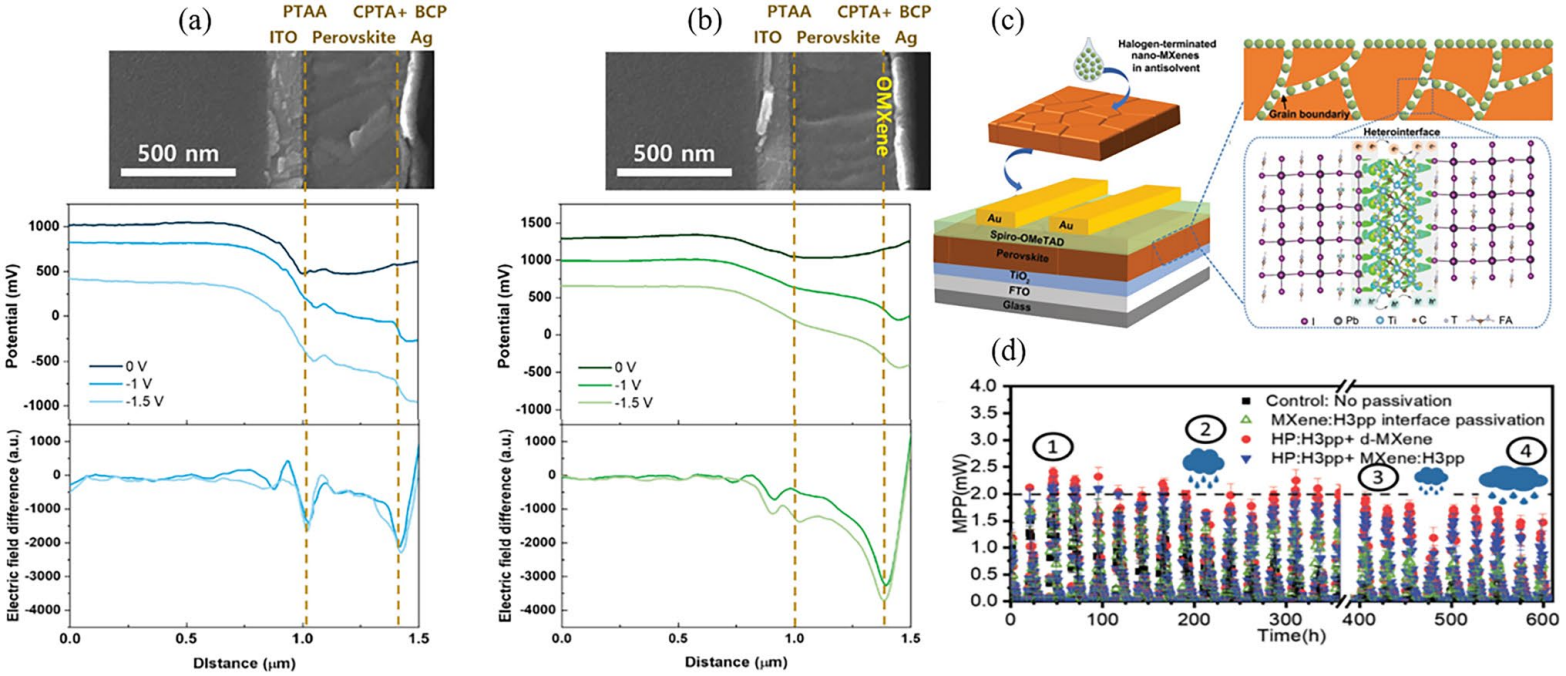
**a, b** Kelvin probe force microscopy for electrical potential and field profiling on the cross-sectional surfaces of PSCs **a** without and **b** with OMXene. Reproduced with permission from Ref. [170]. Copyright 2022, Elsevier. **c** Schematic illustration of PSCs embedding of halogen-terminated nano-MXenes. The functional heterointerface between the perovskite and nano-MXenes is achieved via Pb-T-based ionic lattice anchoring. Reproduced with permission from Ref. [191]. Copyright 2022, Wiley. **d** ISOS-O protocol tracking for PSCs employing bulk and interface passivation. obtained averaged maximum power point values for 3–4 devices for each category during 600 h by holding the encapsulated devices at their open-circuit voltage and recording their *J-V* by 20-min intervals in outdoor at Barcelona, 41.5021°N, 2.1039°E, Spain. Reproduced with permission from Ref. [192]. Copyright 2023, Wiley

Despite the performance enhancement achieved in MXenes-based PSCs, the operational stability of the device is still not satisfactory. To address this issue, nano-MXenes with tailored halogen-terminated surfaces (F_x_, Cl_x_, Br_x_, I_x_) were prepared through pulsed laser irradiation [191]. Halogen anions have the ability to stabilize the soft perovskite lattice, thereby establishing a robust interface between MXenes and perovskite layers (Fig. 10c). This strategy regulates the deep-level defects and WF of the interface, as well as reducing the charge transport barrier. The champion device exhibits a high PCE of 24.17%, and it maintains a consistent performance of over 90% even after operating at the maximum power point for 1,000 h. In addition, it is observed that when Ti_3_C_2_T_x_ is oxidized by NaOH aqueous solution, anatase TiO_2_ nanoparticles can be formed on its surface [170]. The kelvin probe force microscopy results indicate that the oxidized Ti_3_C_2_T_x_ strengthens the electric field at the perovskite/ETL interface and expands the depletion region throughout the perovskite layer (Fig. 10a, b). With such a surface engineering approach, the inverted CsPbI_3_ PSC attains a PCE of 19.69% (0.096-cm^2^) and 14.64% (25-cm^2^ minimodules), respectively. After enduring more than 1,000 h of simultaneous exposure to damp heat (85 °C/85% RH) and intense 1-sun light soaking, the encapsulated minimodule maintains 85% of its initial PCE. Recently, 3-phosphonopropionic acid (H3pp) has been reported as an additive to obtain functionalized MXene Ti_3_C_2_ (MXene:H3pp), which further forms a HP/MXene:H3pp heterojunction with Halide Perovskite (HP). This strategy strengthens the connection between the perovskite layer, the interface, and the Mxene, resulting in an increase in device PCE from 20.56% to ~ 22% [192]. Encouragingly, after outdoor testing (ISOS-O) carried out for > 600 h, the MXene-modified device reveals a *T*_*80*_ of ~ 600 h, while the control device degrades completely (Fig. 10d). This is the first report of the stability assessment of MXene-based PSCs carried out under real outdoor conditions, which pave the way for the commercialization of PSCs.

### Black Phosphorus and Other 2D Materials

**Fig. 11:**
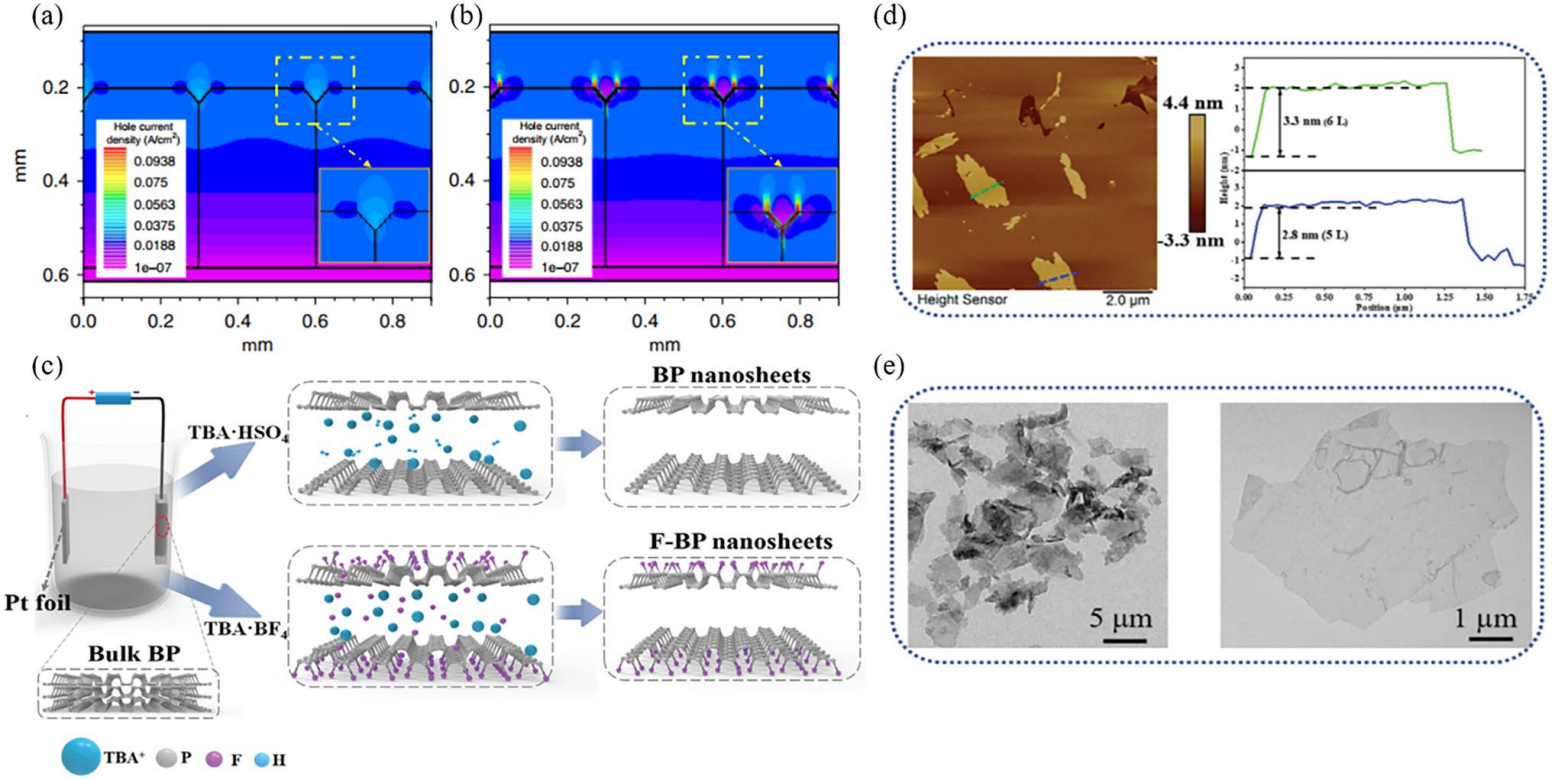
**a, b** The simulated hole current density distribution for the PSCs **a** without and **b** with BP modification. Reproduced with permission from Ref. [194]. Copyright 2021, AAAS. **c** Schematic illustration of the delamination procedure in a two-electrode reaction cell. **d** Atomic force microscope and **e** Transmission electron microscopy images of F-BP nanosheets. Reproduced with permission from Ref. [168]. Copyright 2022, Wiley

The electronic band structure of BP is dependent on its number of layers due to its puckered orthorhombic lattice, quantum confinement effects, and interlayer interactions [43, 100]. This imparts a unique characteristic to the material for interface engineering. For instance, in order to enhance the performance of all-inorganic PSCs with a structure of FTO/c-TiO_2_/CsPbI_3_/CuSCN/Au, different layers of BP were spin-coated between the CsPbI_3_ and the CuSCN HTL [193]. It is revealed that a BP layer count ranging from one to two exhibits a remarkable harmony in energy levels with CsPbI_3_. Moreover, the dielectric shielding effect of BP leads to an improved dissociation efficiency of excitons, resulting in an increase in the carrier density of CsPbI_3_ from 1.92 × 10^14^ to 2.82 × 10^14^ cm^−3^. As a result, the PCE of the all-inorganic PSC enhanced from 10.48% (with *V*_OC_ = 1.02 V, *J*_SC_ = 15.8 mA cm^−2^, FF = 65.1%) to 14.17% (with *V*_OC_ = 1.08 V, *J*_SC_ = 19.3 mA cm^−2^, FF = 68.4%), which was considered as the state-of-the-art performance at that time. The recent studies indicate that BP, with its superior hole mobility (~ 1,000 cm^2^ V^−1^ s^−1^), allows for effective conduction of holes out of the perovskite grain boundaries [194]. This finding confirms that the presence of grain boundaries in perovskite does not have a detrimental effect on the performance device, as long as the accumulated charge carriers within it are promptly evacuated. On the contrary, the combination of grain boundaries and BP creates a fast path for hole transport, resulting in higher hole current density (Fig. 11a, b).

In spite of the impressive performance of BP, the prevailing approach for preparing BP relies on liquid-phase exfoliation assisted by ultrasonic treatment. Unfortunately, this method inevitably introduces impurities and defects that compromise the electronic characteristics of BP. Moreover, the inadequate lateral size of BP can lead to an uneven distribution within interfaces, limiting its full utilization and potential advantages. To effectively tackle these challenges, an innovative synthetic technique through electrochemical delamination has been proposed (Fig. 11c) [168]. This method allows for the achievement of average lateral dimensions of both BP and fluorinated BP (F-BP) at a remarkable magnitude of 4.75 µm, with a thickness spanning only 4–5 atomic layers (Fig. 11d, e). When used as interlayers for hole injection between the perovskite and spiro-OMeTAD, these large-scale, high-quality BP and F-BP nanosheets demonstrate outstanding performance, as the PCE of the device has been enhanced from the initial value of 19.63% (control) to 20.76% (BP) and 22.06% (F-BP), respectively. It is noteworthy that F-BP demonstrates superior stability in comparison to BP. This can be attributed to the formation of hydrogen bonds with MA^+^ and FA^+^ ions, as well as ionic bonding with Pb^2+^ ions, which is facilitated by the presence of F^−^. Additionally, the modification with fluorine enhances the inherent antioxidant and moisture resistance properties of BP.

In addition, antimonene, CNs, MOFs, and 2D perovskite materials have also been reported to be used for the top interface of PSCs [69, 70, 195, 196]. Among them, 2D perovskite materials are particularly noteworthy. Despite exhibiting impressive PCE as a light-absorbing material in photovoltaic devices, the stability of 3D perovskite has remained a persistent challenge [197, 198]. On one hand, the weak hydrogen or ionic bonding between small A cations and corner-sharing BX_6_ octahedra renders 3D perovskite compounds vulnerable upon exposure to moisture or polar solvents. This leads to irreversible decomposition, compromising their stability and lifetime. On the other hand, the fabrication of 3D polycrystalline films through solution processing inevitably gives rise to various inherent defects on their surface. Furthermore, during the crystallization process, intense visible pinholes and cracks commonly emerge. These unavoidable imperfections induce severe charge recombination, resulting in considerable energy loss [32, 35].

In order to deal with these problems, 2D perovskite materials with improved stability and elevated activation energy have garnered significant interest [13, 162, 199]. Unlike their fragile 3D counterparts, 2D perovskites consist of larger amine cations, such as butylamine (BA) and phenethylamine (PEA), which are coordinated within the cages of inorganic BX_6_ octahedra. Due to the large radius of the organic amine cation, the upper and lower inorganic slabs cannot be linked by shared corners, leading to the formation of a 2D structure with alternating organic and inorganic layers [200, 201]. The crystal structure of 2D perovskites can be envisioned as the cleaving of the 3D network of ABX_3_ perovskite along the (100), (110), and (111) planes, resulting in the formation of (100), (110), and (111)-oriented 2D perovskites, respectively [30, 115]. Among these orientations, the (100) has been demonstrated to be the most favorable structure for solar cells. Based on the number of coordinative amino heads present in the spacer cations, this subgroup can be further categorized into three distinct phases: the Ruddlesden–Popper (RP) phase, the Dion–Jacobson (DJ) phase, and the alternating cations in the interlayer space (ACI) phase. Notably, 2D perovskite can be used as a light-absorbing material to constitute photovoltaic devices. Nevertheless, its narrower absorption spectrum and inferior carrier transport efficiency render devices utilizing 2D perovskite less efficient compared to those based on 3D perovskite [202, 203].

By harnessing the complementary advantages of both 3D and 2D perovskites, researchers crafted 2D/3D perovskite heterostructures to achieve the coexistence of high efficiency and ultrastability in PSCs [204–206]. There are primarily two methods for fabricating this stacking-layered architecture: one involves mixing a 2D precursor material with a 3D precursor solution, while the other involves forming a 2D/3D bilayer structure through the reaction of a 2D precursor solution on the surface of a 3D layer. Overall, the combination of 3D and 2D perovskites is achieved through chemical bonding, facilitated by the presence of a pair of non-bonding electrons originating from the N in amines. The merits of 2D/3D heterostructures are as follows [30, 201]: (i) The lattice discrepancies between 2 and 3D perovskites induce surface reconstruction of the 3D material, effectively reducing defects at its grain boundaries and surfaces. Additionally, the sizeable organic iodonium salt simultaneously fills cation/anion vacancies, interacts with under-coordinated lead clusters, and embeds into surface boundaries, pinholes, and cracks. (ii) Compared to 3D perovskites with small A cations, the strengthened molecular interaction between bulky organic cations and inorganic octahedral units contributes to superior photo- and thermal stability. Moreover, the hydrophobic nature of bulky organic cations, coupled with the formation of atomically dense layers, provides a steric hindrance that prevents erosion caused by humidity/oxygen and suppresses ion migration. (iii) 2 and 3D perovskites possess high and low energy levels, respectively. The distinct energy level difference in 2D/3D heterostructures can facilitate carrier extraction and transport.

Currently, remarkable advancements in the performance of PSCs have been realized through the utilization of 2D/3D stacked heterojunctions [196, 207]. For example, the researchers spin-coated pre-synthesized 2D perovskite ((BA)_4_AgBiBr_8_) nanosheets onto the surface of 3D-FAPbI_3_-based perovskite [71]. Due to the vdW force and the large bandgap of (BA)_4_AgBiBr_8_ (2.35 eV), a type-I heterojunction was obtained. This novel contact creates a barrier at the top interface, which inhibits trap-assisted recombination and mitigates the iodide ion diffusion from perovskite to the metal electrode. As a result, a satisfying PCE of 24.48% is achieved with an improved *V*_OC_ from 1.13 to 1.17 V. After continuous irradiation for 1,000 h, the 3D/2D established PSCs retain ~ 90% of their initial PCE. However, the solution-based preparation of 2D perovskites lacks control over phase purity, film thickness, and orientation, which poses challenges for efficient carrier extraction and transport between 3 and 2D perovskites. Therefore, solvent-free preparation techniques have been proposed as a viable strategy. For instance, Noh et al. developed a solid-state in-plane growth (SIG) approach, which facilitates the growth of pure-phase and thickness-tunable BA_2_PbI_4_ films on FA-based 3D perovskite films [208]. The intact 2D/3D halide junction, upon formation, not only extends the carrier lifetime but also establishes an ideal back-surface field for efficient hole transfer. The PSCs with the intact 2D/3D junction demonstrate a remarkably enhanced PCE of 24.59%. Furthermore, these devices maintain an impressive 94% of their initial PCE even after enduring a 1,056-h damp heat test (85 °C/85% RH).

## Graphene Electrodes

The ideal electrode material should possess a commendable level of electrical conductivity, a WF that harmonizes with the neighboring CTLs, exceptional chemical and mechanical stability, all while being cost-effective [22]. Furthermore, for the transparent electrode and back electrode, it is requisite that they exhibit abundant transparency and reflectivity, respectively. Traditional electrode materials utilized in PSCs, including FTO, ITO, Ag, and Au, often suffer from inadequate mechanical and chemical stability, as well as relatively high prices [36, 38]. To address these challenges, 2D graphene has garnered increasing attention in recent years due to its excellent electrical conductivity, high transmittance, large surface area, and stable mechanical properties [37, 60, 209].

### Graphene as Transparent Electrodes

The enthusiasm for exploring 2D graphene as a transparent electrode primarily stems from the necessity in fabricating flexible solar cells. These types of cells find extensive application in powering energy supply systems for wearable electronic devices, the Internet of Things, intelligent communication, portable electronic products, etc. [37, 210–212]. The customary preference for ITO/polyethylene naphthalate (PEN) as the substrate for the fabrication of flexible PSCs is well-documented. Nonetheless, the scarcity of indium, a rare resource, poses challenges for the long-term sustainability and advancement of the photovoltaic industry. In addition, it should be noted that the use of ITO in solar cells is often accompanied by issues such as mechanical instability. According to reports, solar cells using ITO/PEN substrate with an initial PCE of 12.2% could only maintain 50% after undergoing 1,000 bends at a bending radius of 4 mm [213]. In contrast, 2D graphene exhibits exemplary optical and electrical attributes, while maintaining mechanical stability and strong chemical inertness. Furthermore, owing to the abundant reserves of carbon, it emerges as the ideal alternative for fabricating flexible and transparent electrodes.

**Fig. 12:**
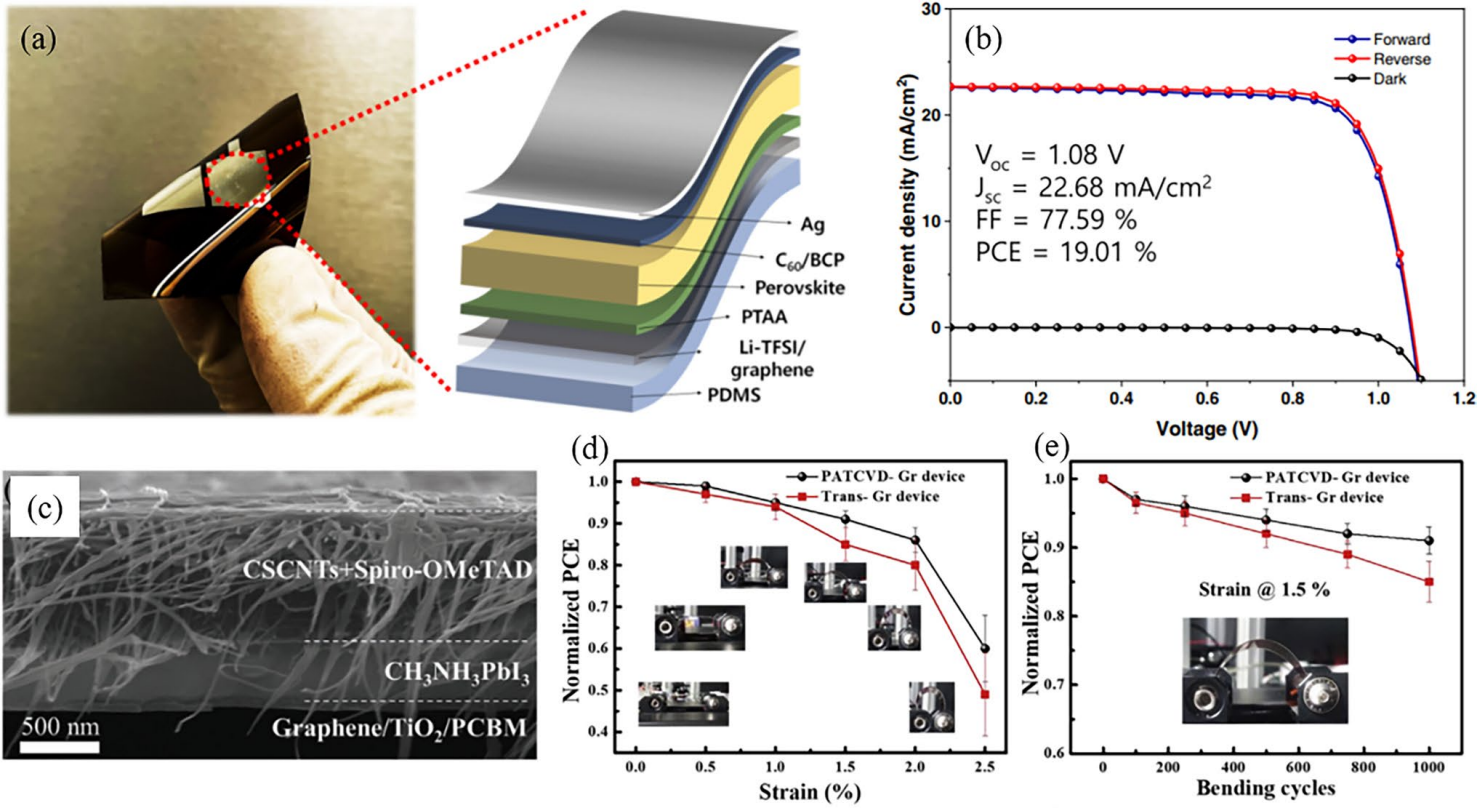
**a** Photograph and schematic illustration of the Li-TFSI-treated graphene/PTAA transparent electrode-based PSCs with an active area of 1 cm^2^ and **b** corresponding *J–V* curves. Reproduced with permission from Ref. [214]. Copyright 2021, Wiley. **c** Cross-sectional scanning electron microscopic image of the all-carbon-electrode-based flexible PSCs. Reproduced with permission from Ref. [212]. Copyright 2018, Wiley. **d, e** Variations in the normalized PCEs **d** measured after 1,000 bending cycles at each tensile strain and **e** as a function of bending cycle under a tensile strain of 1.5%. Reproduced with permission from Ref. [215]. Copyright 2019, Elsevier

Doping plays a crucial role in regulating the WF and series resistance, as well as carrier collection efficiency of graphene electrode. In the past, researchers often adopted AuCl_3_ as a doping agent for graphene [216]. However, the inherent absorption of AuCl_3_ results in certain optical losses, which indicates an urgent need to explore novel dopant. Recently, bis(trifluoromethanesulfonyl)-amide[((CF_3_SO_2_)_2_NH)] (TFSA) has been found to be an effective dopant in graphene [217]. The researchers have uncovered the significance of doping concentration in influencing the optoelectronic characteristics of graphene. It was found that by attaining a concentration of 20 mM in TFSA, the graphene exhibited substantial improvements in both sheet resistance and WF. The original values of these two parameters, 650 for sheet resistance and -4.52 eV for WF, have been adjusted to 116 and -4.90 eV, respectively. On the other hand, there is only a slight decrease in transmittance, with the value shifting from 97.18% to 96.80%. In addition, the incorporation of lithium bis(trifluoromethane)sulfonimide (Li-TFSI) as a dopant has been highlighted for both a single-layer graphene substrate and its neighboring PTAA HTL (Fig. 12a) [214]. Through this modification, a PCE of 19.01% was achieved for a flexible device with an active area of 1 cm^2^ (Fig. 12b), which represents the highest reported PCE among flexible, TCO-free PSCs. Moreover, this type of device demonstrates excellent bending and light stability. After continuous illumination for 1,000 h, the PCE remained at a remarkable 80%.

The versatility of carbon bonds enables carbon materials to function as both p-type and n-type semiconductors, while also exhibiting properties similar to metals [37]. Consequently, there has been a growing interest in the development of all-carbon electrodes, HTL-free, and flexible PSCs. This innovative approach not only presents an alternative opportunity to address the cost and stability concerns associated with photovoltaic technology, but also holds promise for future breakthroughs. For example, a PCE of 8.4% has been achieved in a device with architecture of PET/Graphene/TiO_2_/PCBM/MAPbI_3_/cross-stacking carbon nanotubes (Fig. 12c) [212]. This all-carbon electrode-based, HTL-free PSC presents extraordinary mechanical stability in comparison to ITO-based counterparts. Moreover, it outperforms devices using Au and Ag electrodes in terms of both light soaking and thermal stress resistance. Further investigations have shown that replacing the TiO_2_ ETL with vapor-deposited C_60_ produces a notable reduction in the notorious hysteresis, concurrently achieving a PCE of 13.93% [60].

Despite the remarkable performance exhibited by graphene electrodes, it is important to note that the synthesis of graphene on a large scale requires high temperatures reaching up to 1000 °C [38]. Additionally, when attempting to fabricate flexible PSCs, the transfer of graphene from alternative substrates onto a polymer base can lead to structural nonuniformities in the final samples, including wrinkles, ripples, and lattice defects [218]. These factors impose limitations on the commercialization of graphene electrodes and also have an impact on the fabrication of high-quality PSCs. To address these issues, several novel techniques characterized by low-temperature, non-transferability have been proposed. For instance, the researchers have successfully achieved the direct fabrication of monolayer graphene on flexible polyestersulfone substrates using plasma-assisted thermal chemical vapor deposition (PATCVD) [215]. Notably, this technique necessitates a mere temperature of 150 °C for thin film growth. Following this groundwork, PSCs with a 300 nm-thick perovskite absorber layer have been prepared and yields a PCE of ~ 14.2%. Significantly, even after undergoing 1,000 cycles of bending, these devices maintain a retention rate of 90% of their initial PCE (Fig. 12d, e). Moreover, the interface contact between graphene and adjacent semiconductors can be improved through surface functionalization. As an example, recent findings have shown that surface engineering with ethylene glycol enhances the abundance of surface active sites on graphene, thereby facilitating the deposition of ZnO thin films through atomic layer deposition [219]. This approach reduces interfacial resistance, enhances photon transmission, and consequently results in improved efficiency and stability of the PSCs.

### Graphene as Back Electrodes

There are two key factors driving the research on the utilization of 2D graphene as back electrodes in PSCs: (1) Traditional carbon electrode materials have a porous structure that is characterized by numerous gaps and grain boundaries. These features hinder their ability to establish close contact with the charge transport layers or the perovskite films [220, 221]. (2) With the emergence of semi-transparent solar cells, there is a growing need for back electrodes that combine high conductivity with transparency [222, 223]. Among various options, 2D graphene stands out as an ideal material that satisfies these requirements.

To enhance the interface contact between graphene and perovskite, nitrogen-doped graphene frameworks (N-GFs) have been synthesized via a facile one-step fast pyrolysis [224]. The as-prepared composite material exhibits a large specific surface area (1149 m^2^ g^−1^), which enables a full-cover adsorption with the perovskite layer. These properties make it capable of replacing expensive organic HTL and noble metal electrodes, serving as the top electrode in HTL-free PSCs. Moreover, the introduction of nitrogen atoms provides additional lone pair electrons, which results in localized strain within the hexagonal carbon lattice. Compared to the undoped graphene, N-GFs have improved the *J*_SC_ (18.69 to 20.02 mA cm^−2^) and FF (55.89% to 59.25%) of the HTL-free PSC (FTO/TiO_2_/MAPbI_3_/graphene), resulting in an increase in the PCE from 8.98% to 10.32%. In a recent study, it has been unveiled that the co-doping of graphene with Ni and N (Ni-NG) can decrease the WF of graphene (5.29 to 5.02 eV), thereby enhancing the injection dynamics of holes [225]. Additionally, the presence of Ni atoms on the graphene surface provides an abundance of active sites, which leads to a more intimate interface contact that facilitates the extraction and transport of charge carriers. After implementing this modification, the PCE of PSC with architecture of FTO/SnO_2_/perovskite/Ni-NG/carbon has achieved a noteworthy 12.39%.

**Fig. 13:**
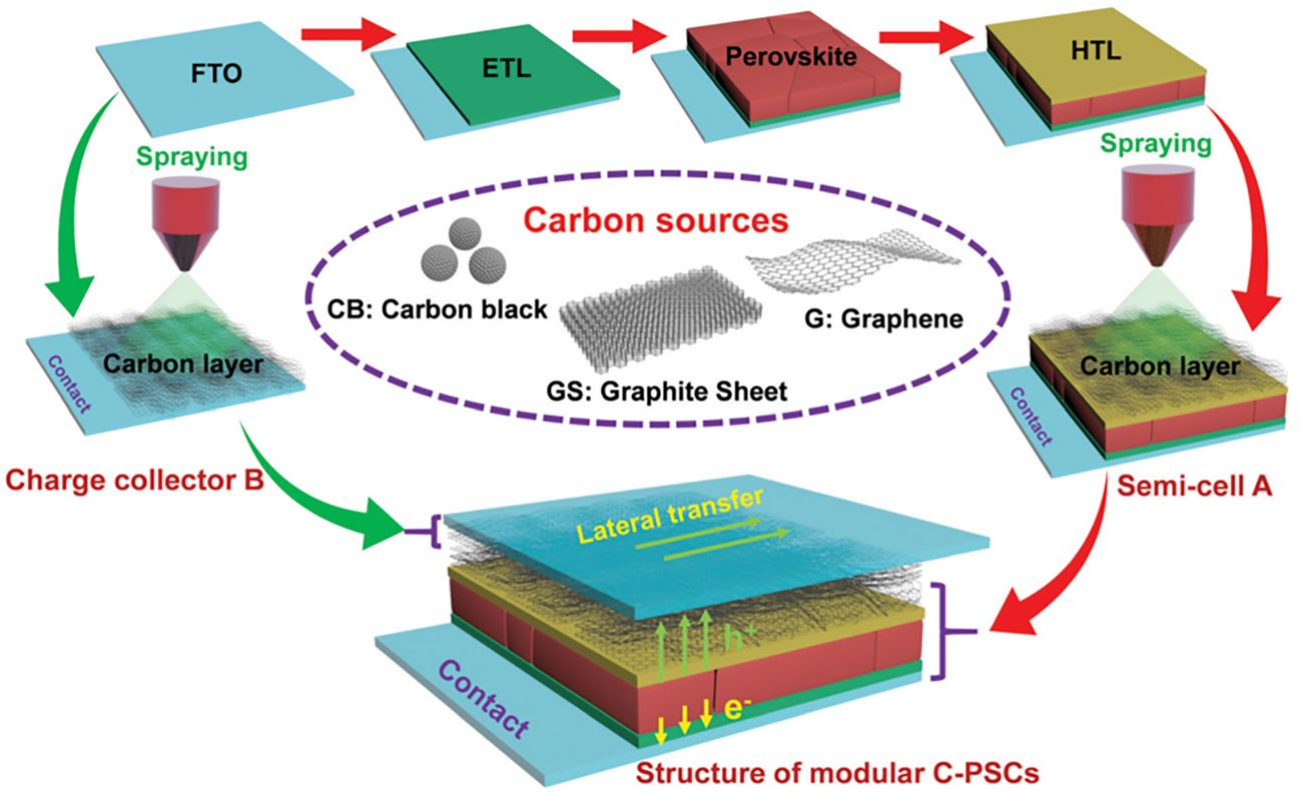
Overview of the fabrication process for the modular C-PSCs; the insets display the microstructure diagrams of the three carbon source materials (CB, GS, and G). Reproduced with permission from Ref. [220]. Copyright 2019, The Royal Society of Chemistry

In order to establish a favorable electrical contact between the carbon electrode and the CTL to minimize sheet resistance and facilitate the large-scale fabrication of the PSCs, an innovative modular approach has been demonstrated. In this scenario, the main body of the solar cell and the carbon top electrode are prepared separately, and subsequently assembled by applying a specific level of pressure (Fig. 13) [220]. The sheet resistance is directly influenced by the magnitude of applied pressure. When the optimal pressure is applied, these two factors create a strong Ohmic contact with minimal series resistance. The authors conducted a comparative analysis of the performance of three carbon electrodes: carbon black, graphite sheet, and 2D graphene. Among them, 2D graphene exhibited the most outstanding overall performance, with a PCE reaching an impressive 18.65%. This achievement stands as one of the highest reported PCE for carbon electrodes in PSCs. Similarly, the researchers first created a hybrid graphene/carbon nanotube (CNT) film using transfer and wet etching techniques [221]. Afterward, they successfully transferred this hybrid film onto the HTL using a lamination process that does not involve water or chemical reactions. In addition to the reduction of resistance, it served as an impermeable shield, safeguarding the interior of the PSCs against the corrosive erosions inflicted by ambient humidity.

## Conclusion and Outlook

In summary, the recent progress of 2D materials in PSCs has been summarized, including graphene and its derivatives, TMDs, MXenes, BP, and others. Due to their unique structures and excellent properties, 2D materials have demonstrated remarkable significance in facilitating the vdW epitaxial growth of perovskites, enhancing charge dynamics, suppressing ion-, moisture-, and oxygen-induced degradation, and serving as electrodes.

Benefiting from these improvements, 2D materials-incorporated PSCs went through rapid advance toward uniting the high efficiency with stability. Notably, a graphene-based PSCs with initial PCE of 24.34% exhibited 5000 h operational stability at the maximum power point under continuous 1-sun illumination and passed the 85 °C/85% RH stability test [36]. In addition, perovskite panels, with total area of 4.5 m^2^ and enhanced by graphene and fMoS_2_, reached a peak power exceeding 250 W under outdoor conditions. As a stand-alone solar farm infrastructure, these panels have been operating for 12 months and achieved a remarkable *T*_*80*_ of 5,832 h [53]. These achievements suggest that the commercialization of PSCs is on the horizon. However, numerous challenges remain to be overcome to bridge the gap between laboratory prototypes and industrialization. The following suggestions are proposed to further accelerate the research of 2D materials in PSCs:

1. Much more efforts are still imperative to deeply understand the underlying physics mechanism of the vdW heterojunction. Due to the distinct bonding characteristics, the growth of perovskite on 2D materials and the resulting interfacial contacts differ significantly from those on conventional substrates. Although vdW epitaxial growth has demonstrated its efficacy in enhancing the grain quality and orientation of perovskite, there remains limited understanding regarding the growth mechanism and the quantitative relationship between the perovskite film and the morphology, size, and thickness of the underlying 2D materials. The theoretical framework is essential to comprehend the nucleation and crystallization processes of perovskite films on 2D materials, as well as the transport mechanism of photogenerated carriers across this heterojunction. The in situ morphological and spectroscopic characterization techniques, such as the integrated differential phase contrast scanning transmission electron microscopy and in situ grazing-incidence X-ray diffraction measurements, may play a pivotal role in elucidating these intricate phenomena [10, 226].
2. The uneven distribution and incomplete coverage of 2D materials at the interface is a great challenge. At present, 2D nanosheets are primarily produced through liquid-phase exfoliation, resulting in the formation of 2D nanosheets with considerable randomness in terms of thickness and shape, and their lateral dimensions are usually restricted to a narrow range of tens to hundreds of nanometers. Hence, it becomes essential to explore fabrication methods that allow for precise control over the morphology and layer number of 2D materials, while simultaneously yielding sizable lateral dimensions and minimized imperfections. In this regard, techniques such as electrochemical deposition and physical vapor deposition present promising avenues to fulfill these aspirations [100, 168].
3. The utilization of 2D materials as electrodes still requires further exploration. The excellent electrical conductivity, mechanical strength, and large surface area of graphene make it an ideal candidate for the transparent and back electrodes. However, several issues must be addressed prior to its commercialization, including the agglomeration, high contact resistance, and the trade-off between high transparency and low sheet resistance. The integration of graphene with metal nanowires or CNTs in the form of a hybrid electrode holds great promise as a future direction. In addition, further exploration should be conducted on other 2D materials that possess the potential to serve as electrodes, such as 2D Ti_3_C_2_ [227–229].
